# PML-Regulated Mitochondrial Metabolism Enhances Chemosensitivity in Human Ovarian Cancers

**DOI:** 10.1016/j.cmet.2018.09.002

**Published:** 2019-01-08

**Authors:** Géraldine Gentric, Yann Kieffer, Virginie Mieulet, Oumou Goundiam, Claire Bonneau, Fariba Nemati, Ilse Hurbain, Graca Raposo, Tatiana Popova, Marc-Henri Stern, Valérie Lallemand-Breitenbach, Sebastian Müller, Tatiana Cañeque, Raphaël Rodriguez, Anne Vincent-Salomon, Hugues de Thé, Rodrigue Rossignol, Fatima Mechta-Grigoriou

**Affiliations:** 1 Institut Curie, Stress and Cancer Laboratory, Equipe Labelisée par la Ligue Nationale contre le Cancer, PSL Research University, 26, rue d’Ulm, 75005 Paris, France; 2 Inserm, U830, 26, rue d’Ulm, Paris 75005, France; 3 Translational Research Department, Laboratory of Preclinical Investigation, Institut Curie, 26, rue d’Ulm, Paris 75248, France; 4 Institut Curie, PSL Research University, CNRS, UMR 144, 75005 Paris, France; 5 Structure and Membrane Compartments, CNRS, UMR 144, 75005 Paris, France; 6 Cell and Tissue Imaging Core Facility PICT-IBiSA, Institut Curie, 75248 Paris, France; 7 DNA Repair and Uveal Melanoma (D.R.U.M.) team, Institut Curie, 26, rue d’Ulm, 75248 Paris Cedex 05, France; 8 Collège de France, PSL Research University, Place Marcellin Berthelot, 75005 Paris, France; 9 Inserm UMR 944, Equipe Labellisée par la Ligue Nationale contre le Cancer, Paris Diderot University, Hôpital St. Louis, Paris, France; 10 Chemical Biology of Cancer, CNRS UMR3666, Inserm U1143, Institut Curie, Equipe Labellisée par la Ligue Nationale contre le Cancer, PSL Research University, 26, rue d’Ulm, Paris 75248, France; 11 Department of Pathology, Institut Curie Hospital, 26, rue d’Ulm, 75248 Paris, France; 12 Inserm U1211, Université de Bordeaux, 146 rue Léo Saignat, 33000 Bordeaux, France

**Keywords:** OXPHOS, reactive oxygen species, promyelocytic leukemia protein, peroxisome proliferator-activated receptor gamma coactivator-1α, PGC-1α, response to treatment, ovarian cancer, oxidative stress, chemoresistance, ferroptosis

## Abstract

High-grade serous ovarian cancer (HGSOC) remains an unmet medical challenge. Here, we unravel an unanticipated metabolic heterogeneity in HGSOC. By combining proteomic, metabolomic, and bioergenetic analyses, we identify two molecular subgroups, low- and high-OXPHOS. While low-OXPHOS exhibit a glycolytic metabolism, high-OXPHOS HGSOCs rely on oxidative phosphorylation, supported by glutamine and fatty acid oxidation, and show chronic oxidative stress. We identify an important role for the PML-PGC-1α axis in the metabolic features of high-OXPHOS HGSOC. In high-OXPHOS tumors, chronic oxidative stress promotes aggregation of PML-nuclear bodies, resulting in activation of the transcriptional co-activator PGC-1α. Active PGC-1α increases synthesis of electron transport chain complexes, thereby promoting mitochondrial respiration. Importantly, high-OXPHOS HGSOCs exhibit increased response to conventional chemotherapies, in which increased oxidative stress, PML, and potentially ferroptosis play key functions. Collectively, our data establish a stress-mediated PML-PGC-1α-dependent mechanism that promotes OXPHOS metabolism and chemosensitivity in ovarian cancer.

## Highlights

• High-grade serous ovarian cancers display OXPHOS metabolic heterogeneity
• High-OXPHOS show high electron transport chain synthesis and respiration rate
• Oxidative stress in high-OXPHOS HGSOC activates PML-PGC-1α and ETC transcription
• High-OXPHOS HGSOCs show enhanced chemosensitivity through oxidative stress and PML

## Graphical Abstract

**Figure:**
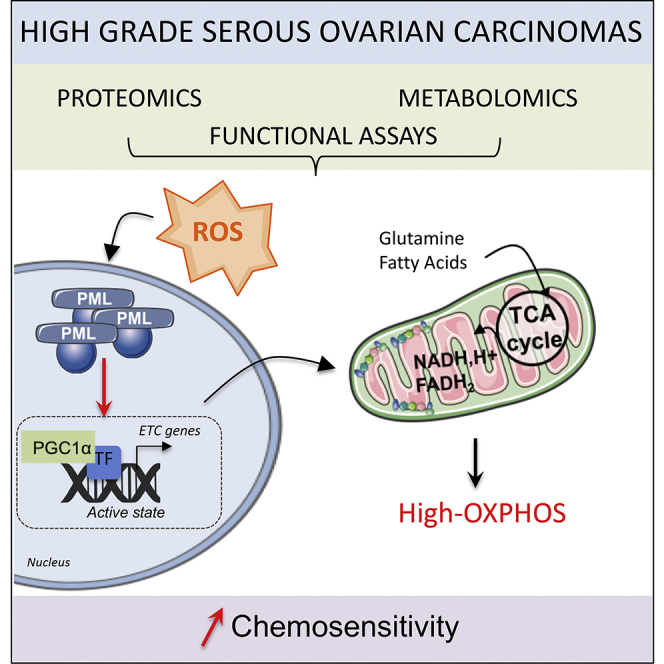

## Teaser

Gentric et al. identify metabolically heterogeneous OXPHOS subgroups in high-grade serous ovarian cancers (HGSOCs). In high-OXPHOS tumors, chronic oxidative stress promotes aggregation of PML-nuclear bodies that activate PGC-1α, electron transport chain synthesis, and mitochondrial respiration. High-OXPHOS HGSOCs exhibit chemosensitivity, in which increased oxidative stress and PML play key functions.

## Introduction

High-grade serous ovarian cancer (HGSOC) remains one of the deadliest gynecologic malignancies and is thus an important clinical challenge. Due to very few early-stage symptoms, ovarian cancers are often diagnosed late, with a subsequent poor prognosis for most patients. To date, treatment strategies mainly rely on the clinicopathologic assessment of tumors and consist of surgery, followed by taxane- and platinum-based chemotherapy. Until now, ovarian carcinomas were mostly classified regarding histologic subtype, grade, and stage. However, recent data based on genomic signatures, i.e., mutations in the *BRCA1* or *BRCA2* genes or methylation of the *BRCA1* or *RAD51C* promoters, lead to homologous recombination deficiency (HRD) and highlight the existence of HGSOC molecular subgroups (Goundiam et al., 2015, Wang et al., 2017). Patients with *BRCA1* or *BRCA2* mutations display an improved response to cisplatin (Cancer Genome Atlas Research Network, 2011, Rigakos and Razis, 2012, Muggia and Safra, 2014, De Picciotto et al., 2016). In addition, transcriptomic profiling allowed the identification of additional HGSOC molecular subtypes (Tothill et al., 2008, Cancer Genome Atlas Research Network, 2011, Mateescu et al., 2011, Bentink et al., 2012, Konecny et al., 2014). One of the first mechanisms identified depends on the miR-200 microRNA and distinguishes two HGSOC subtypes: one related to oxidative stress and the other to fibrosis (Mateescu et al., 2011, Batista et al., 2016).

Metabolic reprogramming has been defined as a key hallmark of human tumors (Gentric et al., 2017, Vander Heiden and DeBerardinis, 2017). But carbon sources in tumors are more heterogeneous than initially thought. Recent studies have revealed the existence of tumor subgroups with a preference for either aerobic glycolysis (typical Warburg effect) or oxidative phosphorylation (OXPHOS) (Caro et al., 2012, Vazquez et al., 2013, Camarda et al., 2016, Hensley et al., 2016, Farge et al., 2017). High-OXPHOS tumors are characterized by upregulation of genes encoding respiratory chain components, together with increased mitochondrial respiration and enhanced antioxidant defense. These metabolic signatures provide important insights into the existing heterogeneity in human tumors. However, this information is lacking with regard to ovarian cancers, and nothing is known about the pathophysiological consequences of metabolic heterogeneity in this disease. Here, our work uncovers heterogeneity in the metabolism of HGSOC and highlights a mechanism linking chronic oxidative stress to the promyelocytic leukemia protein-peroxisome proliferator-activated receptor gamma coactivator-1α (PML-PGC-1α) axis that has a significant impact on chemosensitivity in ovarian cancer.

## Results

### High-Grade Serous Ovarian Cancers Exhibit Metabolic Heterogeneity

**Figure 1:**
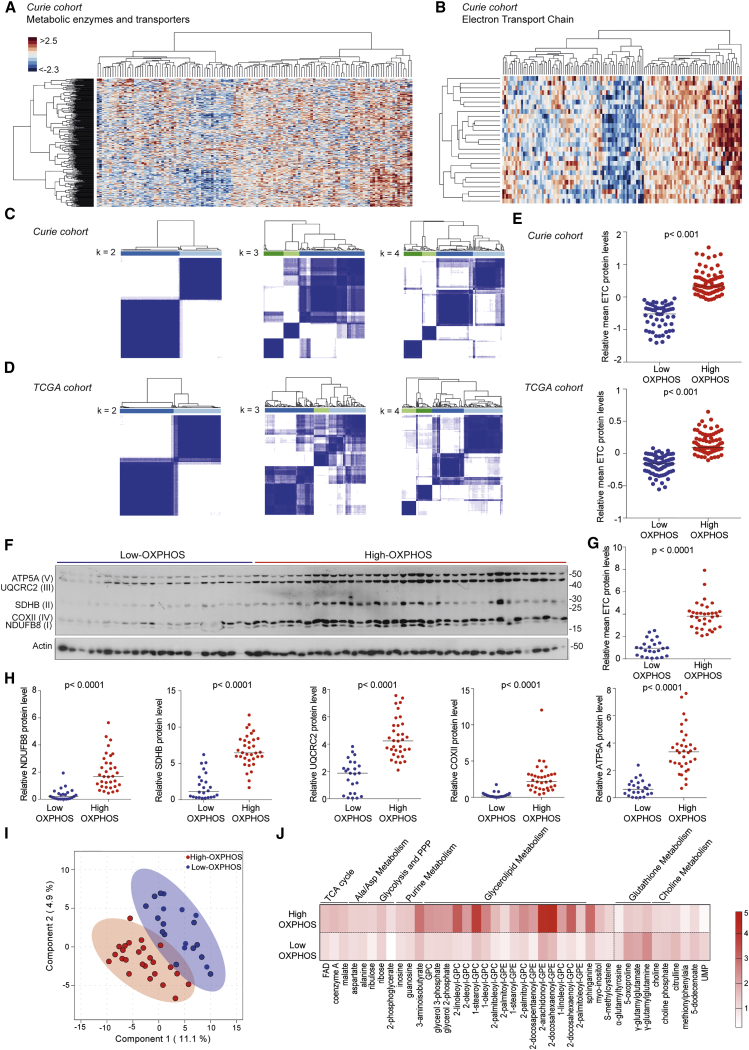
HGSOCs Exhibit Metabolic Heterogeneity (A and B) Hierarchical clustering on 362 metabolic enzymes and transporters (A) and 27 ETC (B) from HGSOC proteomic data (N = 127). Clustering used Ward’s method with Euclidean distance. Each column is a sample; each row a protein. Colors show deviation from the mean (red, above; blue, below). (C and D) Heatmaps showing consensus value matrix from distinct numbers of clusters (*k*) tested, using the *k*-means algorithm. Each row and column represent a sample. Consensus clustering used 1,000 iterations and resampling of 80%. Consensus values are between 0 (white) and 1 (dark blue): 1 means two samples clustered together 100% of times; 0 means they never clustered. Bar plot below the dendrogram shows the consensus clusters. N = 127, Curie (C); N = 169, TCGA (D). (E) Mean of 27 ETC protein levels from proteomic data (Curie, N = 127; TCGA, N = 169). Classification in low- or high-OXPHOS is based on the consensus clustering in (C) and (D). ETC protein levels from Curie have been centered and reduced. Normalization of TCGA data is described in Zhang et al. (2016). Medians are shown. p values from Mann-Whitney test. (F) Representative western blot (WB) showing five ETC proteins (ATP5A, ATP synthase, H^+^ transporting, mitochondrial F1 complex, alpha subunit; UQCR2, ubiquinol-cytochrome *c* reductase core protein II; SDHB, succinate dehydrogenase complex iron sulfur subunit B; COXII, mitochondrially encoded cytochrome *c* oxidase II; NDUFB8, NADH:ubiquinone oxidoreductase subunit B8) in HGSOCs (N = 58). I, II, III, IV, and V indicate ETC complexes. Actin is internal control. (G) Mean of five ETC protein levels quantified from WB as in (F) and normalized to actin. Medians are shown (N = 58; 24 low- and 34 high-OXPHOS HGSOCs). p value from Mann-Whitney test. (H) Same as in (G) for each ETC protein per complex. (I) sPLS-DA of metabolomic data from Curie (N = 45 HGSOCs; n = 374 metabolites). The two clusters were defined with a 95% confidence interval. (J) Heatmap of differential metabolites (t test) between low- and high-OXPHOS HGSOCs (N = 45; n = 41 metabolites). Each column is the mean abundance of each metabolite ranging from white (0) to red (5). Data have been centered and reduced. FAD, flavin adenine dinucleotide; GPC, glycerophosphorylcholine; GPE, glycerophosphoethanolamine; UMP, uridine-2′,3′-cyclic monophosphate. See also Figure S1 and Tables S1, S2, S4, and S5.

To test if HGSOCs show variations in energy metabolism, we first performed a comprehensive label-free proteomic study (Figures 1A–1E) by liquid chromatography-mass spectrometry on 127 HGSOC samples from the Institut Curie cohort (Table S1) and focused our analysis on a list of 360 metabolic enzymes and transporters (Possemato et al., 2011). Hierarchical clustering revealed the existence of at least two HGSOC subgroups with distinct metabolic profiles (Figure 1A). The most differentially expressed metabolic proteins between the two subgroups revealed differences in mitochondrial respiration, electron transport chain (ETC), tricarboxylic acid (TCA) cycle, and ATP biosynthesis process (Table 1). ETC proteins were the most differentially expressed between these two subgroups (Table S2) and could recapitulate these metabolic differences, as shown by restricting our analysis to ETC proteins (Figures 1B and S1A). We also applied a consensus clustering method (Monti et al., 2003) and found that the optimal cluster number of HGSOC subgroups was two (Figure 1C). Importantly, these results were validated in an independent cohort, The Cancer Genome Atlas (TCGA) (Cancer Genome Atlas Research Network, 2011) (Figures 1D and S1B). Here again, classification into two subgroups (hereafter referred to as low- and high-OXPHOS) was the most robust. The consensus clustering-based classification (Figures 1C and 1D) reflected well the mean of ETC protein levels determined by proteomic data (Figure 1E) or by western blots (Figures 1F–1H), thereby demonstrating that this unsupervised classification was appropriate. In addition, the mean level of 27 ETC proteins detected by proteomics was correlated with the 5 ETC proteins analyzed by western blot, particularly in high-OXPHOS HGSOC (Figure S1C), suggesting the level of 5 ETC proteins was sufficient to determine the OXPHOS status. Furthermore, OXPHOS signature (ETC genes listed in Table S3) could also be detected at the transcriptional level in both Curie and TCGA cohorts (Figure S1D).

**Table 1:** Pathways Enriched in the Two Metabolic HGSOC Subgroups

GO Biological Process Term	Count	Percent (%)	Proteins	FDR
GO:0006091∼generation of precursor metabolites and energy	24	48.98	NDUFA5, NDUFB10, NDUFA8, SUCLG2, ALDH5A1, ATP5B, SUCLG1, CYCS, CYC1, ATP5F1, DLAT, OGDH, IDH3A, SDHA, DLD, IDH2, ATP5C1, ATP5L, ATP5O, ATP5A1, ATP5H, ETFB, MDH2, ETFA	3.88E−23
GO:0055114∼oxidation reduction	24	48.98	NDUFA5, HSD17B10, NDUFB10, NDUFA8, ALDH5A1, CYCS, CYC1, GRHPR, DECR1, PRDX3, OGDH, COX5A, HADHA, IDH3A, SDHA, GPX1, DLD, IDH2, SPR, TSTA3, HADH, ETFB, MDH2, ETFA	4.62E−16
GO:0045333∼cellular respiration	12	24.49	SDHA, NDUFA5, NDUFB10, NDUFA8, SUCLG2, ALDH5A1, SUCLG1, CYCS, DLD, IDH2, IDH3A, MDH2	1.71E−11
GO:0006084∼acetyl-CoA metabolic process	8	16.33	SDHA, SUCLG2, SUCLG1, DLD, IDH2, DLAT, IDH3A, MDH2	9.78E−09
GO:0015992∼proton transport	8	16.33	ATP5J2, ATP5B, ATP5F1, ATP5C1, ATP5L, ATP5O, ATP5A1, ATP5H	1.51E−06
GO:0051186∼cofactor metabolic process	10	20.41	SDHA, GPX1, SUCLG2, ALDH5A1, SUCLG1, DLD, IDH2, DLAT, IDH3A, MDH2	1.81E−05
GO:0009109∼coenzyme catabolic process	6	12.24	SDHA, SUCLG2, SUCLG1, IDH2, IDH3A, MDH2	2.89E−05

KEGG Pathways	Count	Percent (%)	Proteins	FDR
hsa05012:Parkinson’s disease	16	32.65	SDHA, NDUFA5, NDUFB10, NDUFA8, SLC25A5, ATP5B, CYCS, CYC1, ATP5C1, ATP5F1, ATP5O, ATP5A1, COX5A, ATP5H, VDAC3, VDAC1	5.26E−11
hsa05016:Huntington’s disease	17	34.69	NDUFA5, NDUFB10, NDUFA8, SLC25A5, ATP5B, CYCS, CYC1, ATP5F1, COX5A, VDAC3, VDAC1, SDHA, GPX1, ATP5C1, ATP5O, ATP5A1, ATP5H	5.16E−10
hsa00190:Oxidative phosphorylation	15	30.61	NDUFA5, ATP5J2, NDUFB10, NDUFA8, ATP5B, CYC1, ATP5F1, COX5A, PPA1, SDHA, ATP5C1, ATP5L, ATP5O, ATP5A1, ATP5H	1.42E−09
hsa00020:Citrate cycle (TCA cycle)	9	18.37	SDHA, SUCLG2, SUCLG1, DLD, IDH2, DLAT, OGDH, IDH3A, MDH2	1.03E−07
hsa05010:Alzheimer’s disease	14	28.57	HSD17B10, NDUFA5, NDUFB10, NDUFA8, ATP5B, CYC1, CYCS, ATP5F1, COX5A, SDHA, ATP5C1, ATP5O, ATP5A1, ATP5H	4.83E−07
hsa00280:Valine, leucine and isoleucine degradation	6	12.24	HSD17B10, DLD, HADH, ACAT1, HADHA, HADHB	0.03054

REACTOME Pathways	Count	Percent (%)	Proteins	FDR
REACT_1505:Integration of energy metabolism	26	53.06	CPT2, ATP5B, CYC1, COX5A, OGDH, ATP5L, ATP5O, ATP5H, ETFB, ETFA, NDUFA5, ATP5J2, NDUFB10, NDUFA8, SLC25A5, SUCLG2, SUCLG1, CYCS, ATP5F1, DLAT, IDH3A, SDHA, DLD, ATP5C1, ATP5A1, MDH2	7.20E−19
REACT_15380:Diabetes pathways	25	51.02	ATP5B, CYC1, COX5A, OGDH, ATP5L, ATP5O, ATP5H, ETFB, ETFA, NDUFA5, ATP5J2, NDUFB10, NDUFA8, SUCLG2, SLC25A5, SUCLG1, CYCS, ATP5F1, DLAT, IDH3A, SDHA, DLD, ATP5C1, ATP5A1, MDH2	1.18E−14
REACT_1046:Pyruvate metabolism and TCA cycle	8	16.33	SDHA, SUCLG2, SUCLG1, DLD, DLAT, OGDH, IDH3A, MDH2	5.95E−06
REACT_1698:Metablism of nucleotides	10	20.41	ATP5J2, SLC25A5, ATP5B, ATP5F1, ATP5C1, AK2, ATP5L, ATP5O, ATP5A1, ATP5H	6.05E−05

Pathway enrichments were defined from Gene Ontology (GO), Reactome, and Kyoto Encyclopedia of Genes and Genomes (KEGG) databases, using DAVID web software (https://david.ncifcrf.gov/). The 50 most differential proteins (from Mann-Whitney test), between the 2 metabolic subgroups of HGSOCs identified in Figure 1A, were used for the enrichment analysis. FDR was computed using the Benjamini-Hochberg procedure to account for multiple testing. REViGO software was used to summarize information by removing redundant GO terms.

We next performed metabolomic analyses on frozen HGSOC samples from the Curie Cohort. Unsupervised analyses on metabolomic data enabled us to confirm the two OXPHOS subgroups of HGSOC (Figure 1I). Differential analysis highlighted the abundance of specific metabolites in each subgroup (Figure 1J). In agreement with increased ETC expression, high-OXPHOS HGSOCs had a significant accumulation of cofactors of oxido-reduction reactions, such as flavine adenine dinucleotide (FAD), coenzyme A (CoA), TCA intermediate (malate), glycerolipid intermediates (ethanolamine and choline family), and metabolites of the pentose phosphate pathway (PPP) (Figure 1J, top). In contrast, low-OXPHOS HGSOCs were characterized by accumulation of glutathione metabolism intermediates (gamma-glutamyl cycle components), as well as choline intermediates (Figure 1J, bottom). Finally, by combining proteomics and metabolomics data, we built a schematic representation of the metabolic pathways that differ between high- and low-OXPHOS HGSOC samples, including OXPHOS, TCA cycle, and fatty acid oxidation (FAO) (Figure S1E). This map illustrated a central role of mitochondrial metabolic pathway reprogramming in HGSOC.

### High-OXPHOS Ovarian Cancer Cells Rely on the TCA Cycle, while Low-OXPHOS Mainly Use Glycolysis

**Figure 2:**
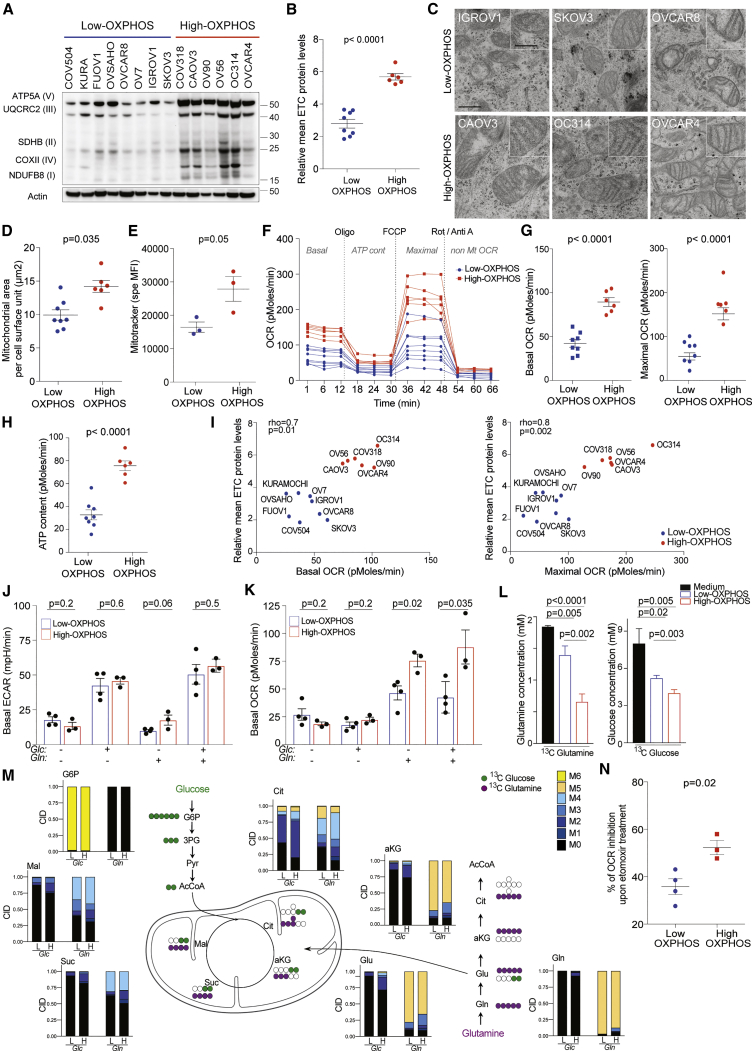
High-OXPHOS Ovarian Cancer Cells Rely on the TCA Cycle (A) Representative WB of five ETC proteins in OCCLs. Actin is internal control. OXPHOS status was defined from quantification in (B). (B) Mean of five ETC protein levels quantified from WB as in (A) and normalized to actin. Data are means ± SEM (n = 3 independent experiments). p value from Student’s t test. (C) Representative electron microscopy (e.m.) pictures from OCCLs. Scale bars, 0.5 μm and 0.25 μm (low and high magnification). (D) Scatterplot showing mitochondrial area per unit of cell surface (in μm^2^) assessed using e.m. of OCCLs listed in (A). Data are means ± SEM (n ≥ 8 e.m. pictures per cell line). p value from Student’s t test. (E) Specific MFI of Mitotracker Deep Red dye in low- (IGROV1, SKOV3, OVCAR8) and high- (OC314, CAOV3, OVCAR4) OXPHOS OCCLs. Data are means ± SEM (n = 3 independent experiments per cell line). p values from Student’s t test. (F) Representative OCR pattern as a function of time (in min), normalized to total protein levels. Oligomycin (Oligo), carbonyl cyanide-4-(trifluoromethoxy)phenylhydrazone (FCCP), rotenone (Rot), and antimycin A (Anti A) were added to measure basal OCR, ATP content, maximal OCR, and non-mitochondrial OCR. N = 14 OCCLs listed in (A). Data are means of four replicates per cell line. (G and H) Basal or maximal OCR (G) and mitochondrial ATP content (H) normalized to total protein levels. N = 14 OCCLs listed in (A). Data are means ± SEM (n ≥ 3 independent experiments). p values from Student’s t test. (I) Correlations between basal (left) or maximal (right) OCR and mean of ETC protein levels. p values are from Spearman test. (J and K) Basal EACR (J) and OCR (K) in presence of 10 mM glucose (Glc) or 2 mM glutamine (Gln), in control conditions (no Glc no Gln or in presence of both 10 mM Glc and 2 mM Gln) in low- (IGROV1, SKOV3, OVCAR8, OV7) and high- (OC314, CAOV3, OVCAR4) OXPHOS OCCLs. Each dot is the mean value for each cell line (n ≥ 3 independent experiments). Bar plots show means ± SEM for each OXPHOS subgroup. p values from Student’s t test. (L) Consumption of [^13^C]-glutamine (left) or [^13^C]-glucose (right) in low- (IGROV1) and high- (OC314) OXPHOS cells after 24 hr of incubation in the corresponding medium. Data are means ± SEM (n = 3 replicates per cell line). (M) Schematic representation of [^13^C]-glutamine- (purple dots) or [^13^C]-glucose-derived carbons (green dots). Bar plots show distribution of isotopologues (M0 to M6 according to labeled carbons) for each metabolite in low- (IGROV1, L) and high- (OC314, H) OXPHOS cells after 24 hr of incubation with -Glc- (10 mM ^13^C-glucose + 2 mM glutamine) or -Gln- (2 mM ^13^C-glutamine + 10 mM glucose). Data are shown as means (n = 3 replicates per cell line). (N) Percentage (%) of OCR inhibition 30 min after etomoxir treatment (40 μM) in presence of 10 mM glucose and 2 mM glutamine in low- (IGROV1, SKOV3, OVCAR8, OV7) and high- (OC314, CAOV3, OVCAR4) OXPHOS OCCLs. Data are means ± SEM (n ≥ 3 independent experiments). p values are from Student’s t test. AcCoA, acetyl coenzyme A; aKG, alpha ketoglutarate; CID, carbon isotopologue distribution; Cit, citrate; Glc, glucose; G6P, glucose 6-phosphate; Glu, glutamate; Gln, glutamine; Mal, malate; Pyr, pyruvate; Suc, succinate; 3PG, 3-phosphoglycerate. See also Figure S2.

Similar to HGSOC samples, two OXPHOS subgroups of ovarian cancer cell lines (OCCLs) were identified using ETC protein levels (Figures 2A and 2B). The subgroup of cells with high-ETC protein levels (called high-OXPHOS by analogy with HGSOC) was characterized by an elevated mitochondrial content (Figures 2C, 2D, S2A, and S2B), mitochondrial network staining (Figure 2E), and mitochondrial transmembrane potential (Figures S2C–S2E). As expected, the mitochondrial area per cell surface unit was strongly correlated with Mitotracker staining in these cells (Rho = 0.94, p = 0.016 by Spearman’s test). High-OXPHOS OCCLs also had a higher oxygen consumption rate (OCR), both at basal and maximal-uncoupled states (Figures 2F and 2G), and exhibited higher mitochondrial ATP content relative to low-OXPHOS cells (Figures 2F and 2H). Basal and maximal OCR were significantly correlated with ETC protein levels (Figure 2I), suggesting a functional association between ETC protein levels and mitochondrial respiration capacity.

We next investigated the carbon sources that fueled the TCA cycle in each OCCL subgroup (Figures 2J–2N). We first observed that both high- and low-OXPHOS OCCLs were able to use glucose to increase extracellular acidification rate (ECAR) (Figures 2J and S2F), but not for OCR (Figures 2K and S2G), indicating that the two subgroups used glucose for glycolysis. In contrast to low-OXPHOS cells, high-OXPHOS OCCLs used glutamine to fuel mitochondrial respiration (Figures 2K and S2G). We also investigated metabolic fluxes by performing isotopic profiling and comparing [^13^C]-glutamine and [^13^C]-glucose use in high- and low-OXPHOS cells. We first observed that high-OXPHOS cells consumed 2.6 times more [^13^C]-glutamine and incorporated 1.4 times more [^13^C]-glucose than low-OXPHOS OCCLs (Figure 2L). Moreover, in [^13^C]-glutamine conditions, there was a decrease in alpha-ketoglutarate (αKG) and citrate M5 isotopologues, and an increase in the M1-M4 isotopologues in high-OXPHOS OCCLs compared to low-OXPHOS cells (Figure 2M), M5 isotopologues coming from cytosolic [^13^C]-glutamine transformation, and M3-αKG and M4-citrate isotopologues resulting from glutaminolysis through TCA cycle. Glutamine anaplerosis in high-OXPHOS was further confirmed by the detection of other labeled TCA compounds, such as succinate and malate (Figure 2M). In [^13^C]-glucose conditions, carbon isotopologue distribution of TCA cycle intermediates confirmed that TCA cycle activity was higher in high-OXPHOS compared to low-OXPHOS cells (Figure 2M). Thus, metabolic fluxes demonstrated that TCA cycle was more active and glutamine more efficiently incorporated in high-OXPHOS than in low-OXPHOS OCCLs. Consistent with these data, glutaminolysis inhibitor reduced OCR in high-OXPHOS OCCLs (Figure S2H). In high-OXPHOS cells, 48% of the citrate came from glutamine and 35% from glucose, suggesting that the 17% left came from another source, such as FAO or pyruvate. This, together with the accumulation of FAO enzymes in high-OXPHOS HGSOCs (Figure S1E; Table S2), prompted us to test the impact of FAO. We couldn’t test the impact of exogenous fatty acids, such as palmitate, because it was highly toxic in OCCLs; thus, we analyzed the impact of FAO inhibition. High-OXPHOS OCCLs were more sensitive to FAO inhibition than low-OXPHOS cells (Figure 2N), suggesting that high-OXPHOS cells also use fatty acids to support mitochondrial respiration. Finally, we tested if these cells could exhibit some metabolic vulnerabilities. Consistent with high-OXPHOS cells relying on active TCA cycle, we found that inhibition of mitochondrial complex I by metformin significantly reduced high-OXPHOS cell viability (Figure S2I) but had no impact on low-OXPHOS cells (Figure S2I).

### High-OXPHOS Ovarian Tumors Exhibit Features of Chronic Oxidative Stress

We next sought to characterize features of OXPHOS heterogeneity in HGSOCs. We first observed that OXPHOS tumors were associated neither with any patient metabolic disorder, nor with tumor properties, such as Ki-67 staining, mitotic index, stage, or debulking efficiency (Figures S3A–S3D). Similarly, there was no significant difference in proliferation, migration, or anchorage-independent growth between low- and high-OXPHOS OCCLs (Figures S3E–S3G). Still, high-OXPHOS cells tend to form fewer colonies than low-OXPHOS cells, consistent with the fact that FAO increases cell survival in conditions of loss of attachment (Schafer et al., 2009) and is required for OCR in high-OXPHOS cells.

**Figure 3:**
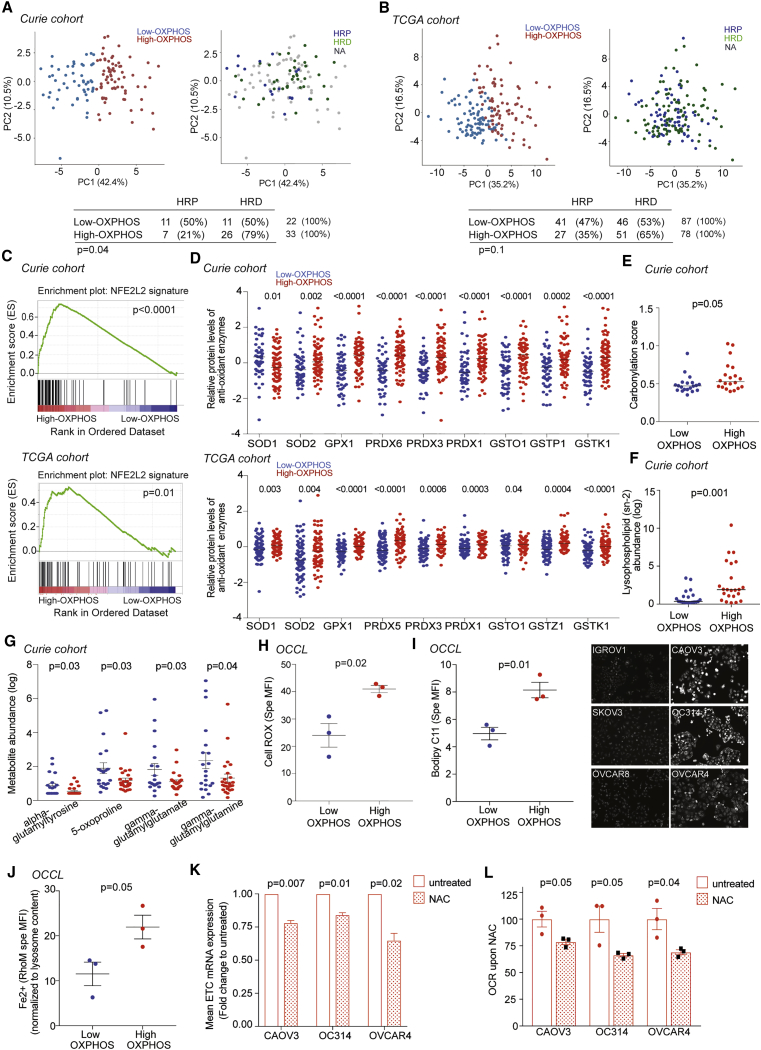
High-OXPHOS HGSOCs Exhibit Features of Oxidative Stress (A) Left: PCA on ETC protein levels (N = 127 HGSOCs; low-OXPHOS, blue, N = 53; high-OXPHOS, red, N = 74). Right: same PCA representation showing HRD (high-LST, green, N = 37) and HRP (low-LST, blue, N = 18) HGSOCs. Unavailable data (NA) are in gray. Bottom: contingency table showing the repartition of low- and high-OXPHOS HGSOCs in HRP and HRD subgroups. p values from Fisher’s exact test. (B) Same as in (A) on TCGA data (N = 169 HGSOCs; low-OXPHOS = 90; high-OXPHOS = 79; HRP = 68; HRD = 97). (C) Gene set enrichment analysis (GSEA) of NFE2L2-target genes in high-OXPHOS HGSOCs (top, Curie; bottom, TCGA). p value from false discovery rate (FDR). (D) Levels of antioxidant enzymes in HGSOCs (top, Curie; bottom, TCGA). Proteomic data are normalized as in Figure 1E. Medians are shown. p values from Mann-Whitney test. (E) Carbonylation scores (carbonylated/total protein levels) in HGSOCs. N = 40. Medians are shown. p values from Mann-Whitney test. (F) sn-2 lysophospholipid abundance (metabolomics data) in HGSOCs. N = 45. p values from Mann-Whitney test. (G) Same as in (F) for gamma-glutamyl intermediates from metabolomic data. N = 45. p values from Student’s t test. (H) Specific MFI using CellROX probe in low- (IGROV1, SKOV3, OVCAR8) and high- (OC314, CAOV3, OVCAR4) OXPHOS OCCLs. Data are means ± SEM (n ≥ 3 independent experiments). p values from Student’s t test. (I) Left: same as in (H) using Bodipy C11 probe. Right: representative views of Bodipy C11 immunofluorescence (IF). (J) Same as in (H) using RhoNox-M (RhoM) probe normalized to the lysosomal content, assessed by lystrocker probe. (K) ETC mRNA levels (ATP5A, UQCRC2, SDHB, COXII, and NDUFB8) in high-OXPHOS OCCLs (CAOV3, OC314, and OVCAR4) treated (red dotted bar) or not (red empty bar) with NAC (5 mM) during 48 hr. Data (fold change normalized to untreated) are means ± SEM (n = 3 independent experiments). p values from one-sample t test. (L) Basal OCR in high-OXPHOS cells (CAOV3, OC314, OVCAR4) upon NAC treatment (5 mM, 48 hr), normalized to the mean of untreated condition for each cell line. Data are means ± SEM (n = 3 independent experiments). p values from Student’s t test. See also Figure S3 and Tables S1 and S3.

As OXPHOS signature was observed in OCCLs, we considered that it was mainly driven by cancer cells and not by stroma. We thus tested the association between OXPHOS and genomic alterations, i.e., DNA copy number alterations (CNAs). While ETC mRNA levels were higher in high- versus low-OXPHOS HGSOCs (Figure S1D), no gene amplification was found in ETC genes or in ETC-regulated transcription factors (Figures S3H and S3I). Moreover, there was no association between OXPHOS stratification and global mutation counts or CNA per tumor (Figure S3J). We also tested the link between OXPHOS and HR status by using the LST (large-scale state transitions) signature, a robust indicator of HRD (Popova et al., 2012, Goundiam et al., 2015), as confirmed by two other published signatures (Abkevich et al., 2012, Birkbak et al., 2012) (Figure S3K). OXPHOS signature was significantly associated with HRD in Curie cohort, with a similar tendency, but not significant, in TCGA (Figures 3A and 3B).

As HRD is known to be associated with chronic oxidative stress (Martinez-Outschoorn et al., 2012, Gorrini et al., 2013), we next evaluated features of oxidative stress in HGSOCs (Figures 3C–3J). We first observed that the NFE2L2 (NRF2)-dependent antioxidant response (list in Table S3) was upregulated in high-OXPHOS tumors in both Curie and TCGA cohorts (Figure 3C) in the absence of any deleterious mutations of *KEAP1* (Kelch-like ECH-associated protein 1). Levels of antioxidant enzymes were also significantly increased in high- compared to low-OXPHOS HGSOCs (Figure 3D), suggesting they suffer from oxidative stress. High-OXPHOS HGSOCs indeed exhibited more oxidized proteins (Figures 3E and S3L) and lipid oxidation products, such as lysophospholipids with acyl chains at sn-2 position (Figure 3E), together with fewer glutathione intermediates (Figure 3G) than low-OXPHOS tumors. Although it was not possible to measure reactive oxygen species (ROS) levels in tumors due to their short half-life, we confirmed that both ROS and lipid peroxidation levels were higher in high- than in low-OXPHOS OCCLs (Figures 3H and 3I). Finally, we determined that high-OXPHOS cells exhibited a higher lysosomal Fe^2+^ content (Figure 3J) than low-OXPHOS cells, thereby confirming that high-OXPHOS cells suffer from chronic oxidative stress. To determine if oxidative stress could be the cause rather than the consequence of high-OXPHOS status, we investigated the impact of antioxidant treatment (N-acetyl cysteine, NAC) on ETC expression and OCR capacity of high-OXPHOS cells. We found that NAC treatment reduced ETC gene expression (Figure 3K) and OCR (Figure 3L), and thus reversed at least in part the high-OXPHOS status. In conclusion, high-OXPHOS cells are characterized by a chronic oxidative stress and this stress is required for high-OXPHOS properties.

### PML-Nuclear Bodies Accumulate in High-OXPHOS HGSOCs and Play a Key Role in OXPHOS Signature through PGC-1α Regulation

**Figure 4:**
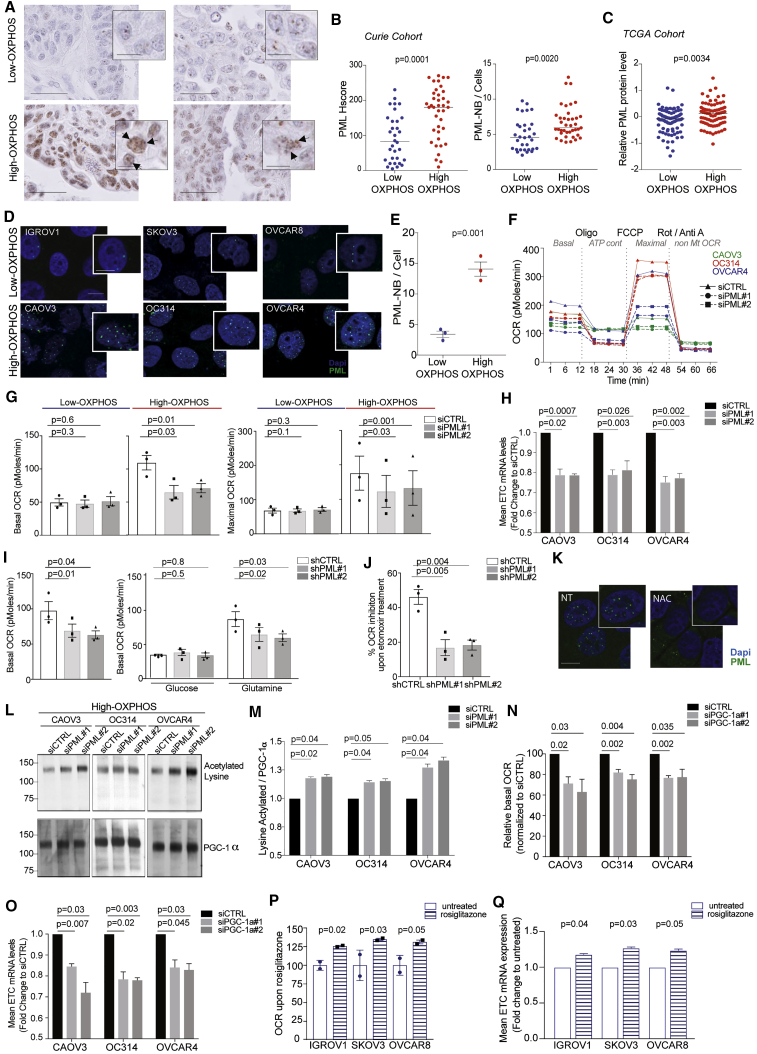
PML Is a Key Actor in High-OXPHOS Ovarian Cancer (A) Representative views of PML IHC in HGSOCs, with PML-NBs (arrows). Scale bars, 50 μm and 10 μm (low and high magnification). (B) PML Hscore (left) and number of PML-NBs per cell (right) in HGSOCs. N = 71. Medians are shown. p values from Mann-Whitney test. (C) PML protein levels in HGSOCs from TCGA cohort (N = 169). Medians are shown. p values from Mann-Whitney test. (D) Representative views of PML IF in OCCLs, with PML-NB (high magnification). Scale bars, 50 μm and 10 μm (low and high magnification). (E) Number of PML-NBs per cell in low- (IGROV1, SKOV3, OVCAR8) and high- (CAOV3, OC314, OVCAR4) OXPHOS OCCLs. Data are means ± SEM (n = 3 independent experiments). p values from Student’s t test. (F) Representative pattern of OCR as a function of time (min) normalized to total protein levels. High-OXPHOS OCCLs (CAOV3, OC314, and OVCAR4) were transfected with non-targeting small interfering RNA (siRNA) (siCTRL) or with two different siRNA targeting PML (siPML#1 and siPML#2). (G) Basal (left) and maximal (right) OCR following PML silencing in low- (IGROV1, SKOV3, OVCAR8) and high- (OC314, CAOV3, OVCAR4) OXPHOS OCCLs. Each dot is the mean value for each cell line (n ≥ 3 independent experiments). Bar plots show means ± SEM for each OXPHOS subgroup. p values from paired t test. (H) ETC mRNA levels (ATP5A, UQCRC2, SDHB, COXII, and NDUFB8) in high-OXPHOS OCCLs (CAOV3, OC314, and OVCAR4) transfected with non-targeting siRNA (siCTRL) or with two siRNA targeting PML (siPML#1 and #2). Data (fold change normalized to non-targeting siRNA) are means ± SEM (n ≥ 3 independent experiments). p values from one-sample t test. (I) Basal OCR in high-OXPHOS OCCLs (CAOV3, OC314, and OVCAR4) transfected with shCTRL, shPML#1 or shPML#2 in presence of 10 mM glucose and 2 mM glutamine (left), 10 mM glucose (middle), or 2 mM glutamine (right). Each dot is the mean value for each cell line (n = 3 independent experiments). Bar plots show means ± SEM of the three cell lines per condition. p values from Student’s t test. (J) Same as in (I). Bar plot shows percent (%) of OCR inhibition, 30 min after etomoxir treatment (40 μM) in presence of 10 mM glucose and 2 mM glutamine. (K) Representative views of PML IF in high-OXPHOS OCCLs (CAOV3) following NAC treatment (5 mM, 48 hr). High-magnification views show PML-NBs. Scale bars, 50 μm and 10 μm (low and high magnification). (L) Representative WB showing acetylated PGC-1α after PGC-1α immunoprecipitation (top) and total PGC-1α protein (bottom) from high-OXPHOS OCCLs (CAOV3, OC314, and OVCAR4) transfected with siCTRL, siPML#1, or siPML#2. (M) Ratio of acetylated PGC-1α to total PGC-1α protein levels upon PML silencing, as shown in (L). Data are means ± SEM of fold changes normalized to siCTRL (n = 3 independent experiments). p values from one sample t test. (N) Basal OCR following PGC-1α silencing (siPGC-1α#1 and #2) normalized to siCTRL in high-OXPHOS OCCL (CAOV3, OC314, and OVCAR4). Data are means ± SEM (n = 3 independent experiments). p values from one-sample t test. (O) As in (H) after transfection with siCTRL, siPGC-1α#1, or siPGC-1α#2. Data (fold change normalized to non-targeting siRNA) are means ± SEM (n = 3 independent experiments). p values are from one-sample t test. (P) Basal OCR in low-OXPHOS cells (IGROV1, SKOV3, and OVCAR8) upon rosiglitazone treatment (10 μM, 48 hr) normalized to the mean of untreated condition for each cell line. Data are means ± SEM (n = 2 independent experiments). p values from one-sample t test. (Q) As in (H) in cells in low-OXPHOS OCCLs (IGROV1, SKOV3, and OVCAR8) treated (stripped bar) or not (empty bar) with rosiglitazone (10 μM, 48 hr). Data (fold change normalized to untreated) are means ± SEM (n = 2 independent experiments). p values from one-sample t test. See also Figure S4.

We next aimed to determine the molecular players involved in OXPHOS regulation downstream of oxidative stress in HGSOCs. There is long-lasting evidence showing that ROS exert active signaling activities (Gentric et al., 2017). Among them, PML factor is a well-known target of oxidative stress; its aggregation is regulated by ROS (Sahin et al., 2014, Tessier et al., 2017). We compared PML protein levels and nuclear bodies (NBs) in low- and high-OXPHOS HGSOCs by immunohistochemistry (IHC) (Figures 4A, 4B, and S4A). High-OXPHOS HGSOCs showed a stronger PML histologic score (Hscore) (Figures 4A and 4B, left panel) and a higher content in PML-NBs per cell (Figures 4A, 4B, right panel, and S4B) than low-OXPHOS tumors. As PML Hscore, PML-NBs, and PML mRNA levels were correlated in HGSOCs of the Curie cohort (Figures S4C and S4D), we tested PML regulation in the TCGA cohort. We found that PML protein (Figure 4C) and mRNA (Figure S4E) levels were also significantly higher in high-OXPHOS HGSOCs than in low-OXPHOS tumors in TCGA, thereby showing the link between PML and OXPHOS status in two independent HGSOC cohorts.

High-OXPHOS OCCLs also had a higher content in PML-NBs than low-OXPHOS cells (Figures 4D and 4E), consistent with their higher ROS content (Figure 3I). Notably, PML silencing (Figures S4F–S4H) decreased both basal and maximal OCR in high-OXPHOS cells, with almost no impact on low-OXPHOS cells (Figures 4F and 4G). In addition, PML silencing in high-OXPHOS OCCLs also reduced expression of ETC genes (Figure 4H). We validated the long-term impact of PML silencing (Figure S4I) on OCR capacity and ETC expression in high-OXPHOS cells using stable cell lines (Figures 4I, left panel, 4J, and S4J), but with no impact on cell proliferation and migration (Figures S4K and S4L). Moreover, incorporation of glutamine to fuel TCA cycle was reduced in PML-silenced high-OXPHOS cells, while glucose-dependent OCR remained low and did not vary (Figure 4I, middle and right panels). In addition, PML silencing in high-OXPHOS cells significantly reduced the impact of FAO inhibitor on OCR (Figure 4J), suggesting that PML was essential for glutamine anaplerosis and FAO in high-OXPHOS cells. Finally, NAC antioxidant treatment, which reduced expression of ETC encoding genes (Figure 3K), also reduced PML-NBs in high-OXPHOS cells (Figure 4K), thereby confirming the important role of ROS in these cells. Thus, PML and ROS are not only associated with, but also necessary for, high-OXPHOS status in HGSOCs.

ETC genes are upregulated at the transcriptional level by PGC-1α, which is itself activated by lysine deacetylation (Rodgers et al., 2005, Tan et al., 2016). As PML was recently identified as an upstream activator of PGC-1α in breast cancers (Carracedo et al., 2012), we tested the impact of PML silencing on PGC-1α in high-OXPHOS OCCLs. PML silencing had no impact on PGC-1α mRNA and protein levels (Figures S4M and S4N), but it increased PGC-1α acetylation on lysine residues, thereby reducing its transcriptional activity (Figures 4L and 4M). As observed for PML, PGC-1α silencing reduced respiration capacities (Figure 4N) and ETC expression (Figure 4O). Interestingly, promoters of the top 50 most upregulated genes in high-OXPHOS tumors were enriched in PPARγ-binding motifs (Enrichr software, http://amp.pharm.mssm.edu/Enrichr/), consistent with the fact that PGC-1α is a co-activator of PPARγ. In contrast, other expected binding sites, such as ERRα, were not enriched. We thus tested whether treatment of low-OXPHOS OCCLs by rosiglitazone, known to increase mitochondrial component biogenesis through activation of PGC-1α and PPARγ (Ohno et al., 2012), could promote high-OXPHOS status. Rosiglitazone indeed increased basal OCR (Figure 4P) and enhanced ETC expression (Figure 4Q) in low-OXPHOS cells, suggesting that mitochondrial biogenesis in low-OXPHOS could be sufficient for establishing high-OXPHOS status. Taken as a whole, these data suggest that PML plays a critical role in OXPHOS metabolism in HGSOCs by modulating PGC-1α transcriptional activity and subsequently ETC gene expression and mitochondrial respiration.

### High-OXPHOS Metabolism Is Associated with Better Prognosis in HGSOC Patients

**Figure 5:**
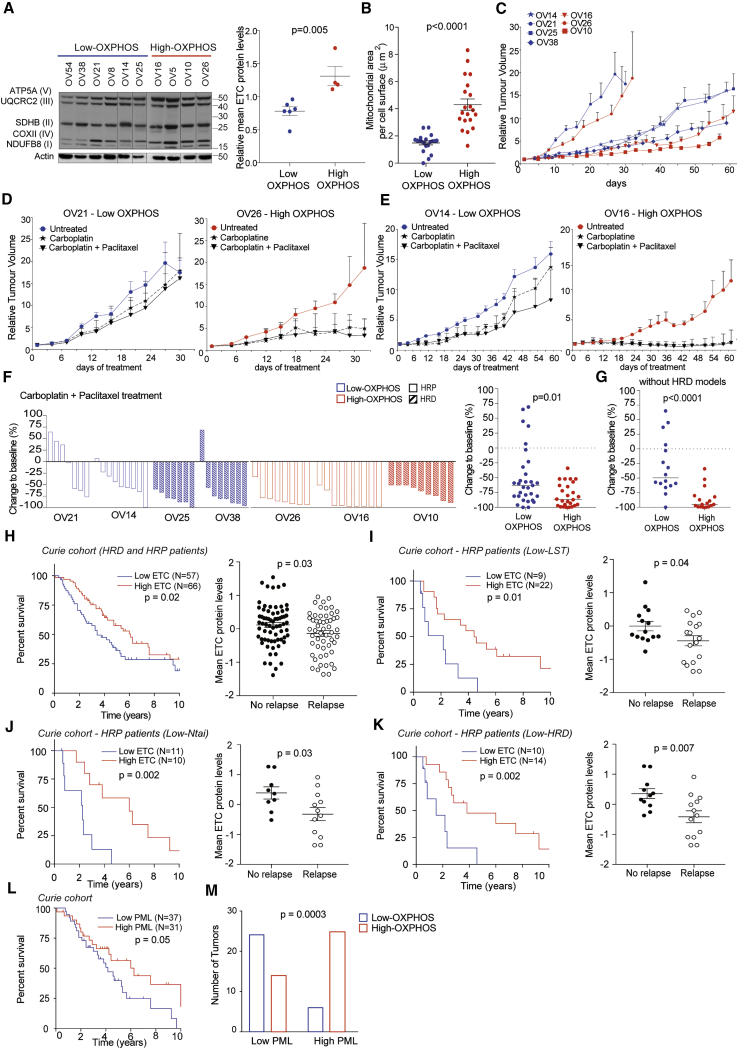
High-OXPHOS Metabolism Is Associated with Better Patient Prognosis (A) Left: representative WB showing five ETC proteins in HGSOC PDX. Actin is internal control. Right: mean of five ETC proteins levels normalized to actin. Data are means ± SEM (N = 10 PDX models). p value from Student’s t test. (B) Mitochondrial area per unit of cell surface (in μm) in low- (OV21) and high- (OV26) OXPHOS PDX. Data are means ± SEM (n = 20 e.m. pictures). p value from Student’s t test. (C) Tumor growth curves (relative tumor volumes [RTV] = *V*_*n*_/*V*0 as a function of time [days]) in low- (blue) and high- (red) OXPHOS HGSOC PDX (low-OXPHOS HRP, OV14, OV21; low-OXPHOS HRD, OV25, OV38; high-OXPHOS HRP, OV16, OV26; high-OXPHOS HRD, OV10). Data are means ± SEM (n ≥ 7 mice per group). (D and E) Tumor growth curves of representative fast- (D) and slow- (E) growing PDX. Mice were treated with NaCl 0.9% (untreated), carboplatin, or carboplatin + paclitaxel, as indicated. Data are means ± SEM (n ≥ 7 mice per group). (F) Left: waterfall plots showing change to baseline (%) per mouse at the end of carboplatin + paclitaxel treatment in each PDX model. Baseline is the mean of untreated control mice. Change to baseline is calculated as (RTV from carboplatin + paclitaxel-treated mice/RTV from control mice) − 1 × 100. Right: change to baseline comparing low- and high-OXPHOS PDX. Medians are indicated. p value from Mann-Whitney test. (G) As in (F), right, restricted to HRP PDX, i.e., low- (OV21 and OV14) and high- (OV26 and OV16) OXPHOS HRP PDX. Medians are indicated. p value from Mann-Whitney test. (H) Left: Kaplan-Meier curves showing 10-year overall survival of HRD and HRP patients with low- (blue) or high- (red) ETC protein levels. p value from log-rank test. Right: mean ETC protein levels in HGSOCs according to relapse status at 12 months after the end of the first line of chemotherapy. Data are means ± SEM. p value from Student’s t test. (I–K) Same as in (H) for HRP patients identified by LST signature (I), N_tai_ score (J), or HRD score (K). (L) Same as in (H) according to PML HScore. p value from log-rank test. (M) Association between low- and high-PML HGSOCs (PML Hscore) and OXPHOS status. p value from Fisher’s exact test. For Kaplan-Meier analyses, stratification of patients was performed using successive iterations to find the optimal sample size thresholds. See also Figure S5 and Table S1.

The association of high-OXPHOS metabolism with tumor response to treatment is still debated (Obre and Rossignol, 2015, Gentric et al., 2017). In order to study the impact of OXPHOS stratification in response to standard chemotherapy (i.e., platinum salts and taxane) in HGSOCs, we took advantage of the ovarian patient-derived xenograft (PDX) mouse models that recapitulate histopathological and molecular properties of the patient’ tumors from which they are derived, as shown previously (Gruosso et al., 2015), including HRD status and response to chemotherapy (Table S1). We first determined the OXPHOS status of ovarian PDX by analyzing ETC protein levels (Figure 5A) and confirmed that high-OXPHOS PDX exhibited a higher mitochondrial area than low-OXPHOS models (Figure 5B). PDX models were screened for their engraftment capacity, and tumor growth of three high- and four low-OXPHOS PDX was analyzed. Although PDX exhibited distinct tumor growth kinetics, we could not detect any difference related to OXPHOS status (Figure 5C). In contrast, chemotherapy responses were different depending on OXPHOS status (Figures 5D–5F and S5A). Tumor growth was more efficiently inhibited upon chemotherapy in high- versus low-OXPHOS PDX models in both fast- and slow-growing tumors (Figures 5D and 5E). Moreover, in two different treatment conditions (carboplatin or carboplatin plus paclitaxel), tumor growth inhibition per mouse was significantly better in high-OXPHOS PDX models (Figures 5F and S5A). Importantly, treatment response was not dependent on the HR status. Indeed, HRD and HRP PDX models were equally distributed in the two OXPHOS subgroups (Figures 5F and S5A). Moreover, when restricted to HRP, high-OXPHOS PDX still showed stronger tumor growth inhibition than low-OXPHOS models (Figures 5G and S5B). Thus, OXPHOS status is associated with better response to chemotherapy in PDX, even in HRP models.

Consistent with these observations on PDX, high-ETC protein level (defining high-OXPHOS status) was associated with improved patient survival (Figure 5H). As progression-free survival reflects both response to chemotherapy and debulking efficiency for most patients, we performed analyses at short term after the first line of chemotherapy (relapse at 12 months) and found that patients with high-ETC protein levels were associated with absence of relapse at 12 months (Figure 5H). Similar results were obtained at mRNA levels (Figure S5C) and when HGSOC patients were stratified according to both HR and OXPHOS status (Figure S5D). Although we did not detect any impact of OXPHOS status on HRD patients, HRP patients (defined either by LST signature, N_tAi_ [Birkbak et al., 2012] or HRD score [Abkevich et al., 2012]) with high-ETC protein levels survived longer and were associated with a lack of relapse at 12 months (Figures 5I–5K) compared to those with low-ETC protein levels. Finally, stratification of HGSOCs into two subgroups according to PML HScore was indicative of patient survival. High-PML HScores were indeed associated with better patient prognosis (Figure 5L) and enriched in high-OXPHOS tumors (Figure 5M), consistent with the role of PML in OXPHOS status. Unfortunately, PGC-1α was not detected in transcriptomic or proteomic data in human samples, thereby precluding the same type of analyses with PGC-1α. Thus, these data confirmed the link between PML and OXPHOS status and its impact on patient survival.

### High-OXPHOS Metabolism Enhances Chemosensitivity by Modulating ROS Levels

**Figure 6:**
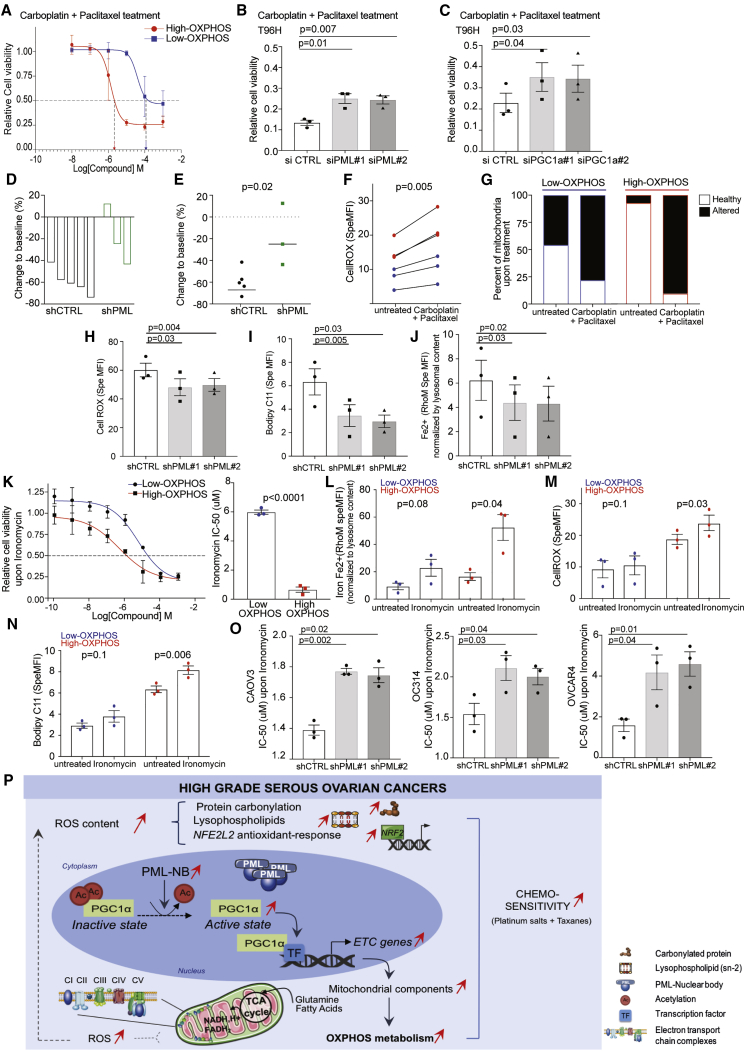
High-OXPHOS Metabolism Enhances Chemosensitivity by Modulating ROS Levels For a Figure360 author presentation of Figure 6, see https://dx.doi:10.1016/j.cmet.2018.09.002#mmc5. (A) Representative dose-response curve showing variation in cell viability of low- (IGROV1, SKOV3, OVCAR8) and high- (CAOV3, OC314, OVCAR4) OXPHOS OCCLs after 48 hr of treatment. Cells were exposed to carboplatin + paclitaxel at concentrations of 0.01 to 1,000 μM. Data relative to vehicle-treated controls are means ± SEM (n = 3 independent experiments). Note that IC_50_^high-OXPHOS^ = 3.5 μM, IC_50_^low-OXPHOS^ = 13 μM. p value from Student’s t test. (B and C) Relative cell viability of high-OXPHOS OCCLs (CAOV3, OC314, and OVCAR4) transfected with non-targeting siRNA (siCTL), siRNA targeting PML (siPML#1 and #2) (B), or siRNA targeting PGC-1α (siPGC-1α#1 and #2) (C). Cells were exposed to carboplatin [5.10^−5^ M] and paclitaxel [10^−6^ M] during 96 hr. Data relative to vehicle-treated controls are means ± SEM (n = 3 independent experiments). p values from paired t test. (D) Waterfall plot showing change to baseline per mouse at the end of carboplatin + paclitaxel treatment in mice engrafted with high-OXPHOS (OC314) stable cell lines expressing either non-targeting (shCTRL) or PML-targeting shRNA (shPML). Baseline is the mean of untreated control group of mice. Change to baseline is calculated as (RTV from carboplatin + paclitaxel treated mice/RTV from control mice) − 1 × 100. (E) Change to baseline comparing shCTRL and shPML mouse models. Medians are indicated. p value from Mann-Whitney test. (F) Specific MFI of CellROX probe in low- (IGROV1, SKOV3, OVCAR8, blue) and high- (CAOV3, OC314, OVCAR4, red) OXPHOS OCCLs following carboplatin + paclitaxel treatment ([5.10^−5^ M] carboplatin+[10^−6^ M] paclitaxel, 24 hr). Data are means ± SEM (n = 3 independent experiments). p values from paired t test. (G) Percent (%) of healthy (white) or altered (black) mitochondria morphology following carboplatin + paclitaxel treatment in low- (OV21) and high- (OV26) PDX models. (n ≥ 9 e.m. pictures). (H) Specific MFI using CellROX probe in high-OXPHOS cells (CAOV3, OC314, and OVCAR4) transfected with shCRTL, shPML#1, or shPML#2. Each dot is the mean value for each cell line (n = 3 independent experiments). Bar plots show means ± SEM of the three cell lines per condition (shCTRL, shPML#1, and shPML#2). p values from paired t test. (I) Same as in (H) using Bodipy C11 probe. (J) Same as in (H) using RhoM probe normalized to the lysosomal content, assessed by lystrocker probe. (K) Left: representative dose-response curve showing variation in cell viability of low- (IGROV1, SKOV3, OVCAR8) and high- (CAOV3, OC314, OVCAR4) OXPHOS OCCLs after 72 hr of ironomycin from 0.0001 to 1,000 μM. Data relative to vehicle-treated controls are means ± SEM (n = 3 independent experiments). Right: bar plot showing the corresponding ironomycin IC50 values (n = 3 independent experiments). p values from Student’s t test. (L–N) As in (H)–(J) in low- (IGROV1, SKOV3, OVCAR8) and high- (CAOV3, OC314, OVCAR4) OXPHOS cells upon ironomycin treatment (6 μM, 24 hr). Data are means ± SEM (n = 3 independent experiments). p values from paired t test. (O) Ironomycin IC50 values in high-OXPHOS cells, CAOV3 (left), OC314 (middle), and OVCAR4 (right) transfected either with non-targeting shRNA (shCTRL) or with shRNA targeting PML (shPML#1 and #2). Data are shown as mean ± SEM (n = 3 independent experiments). p values from paired t test. (P) Around half of HGSOCs are characterized by elevated levels of carbonylated proteins and lysophospholipids, with decreased abundance of glutathione intermediates, all hallmarks of redox imbalance. Oxidative stress promotes PML and PML-NB accumulation, leading to PGC-1α activation through its deacetylation. PGC-1α activation in turn increases transcription of ETC components, further enhancing mitochondrial respiration. High-OXPHOS HGSOCs rely on OXPHOS, as well as glutamine- and fatty acid-fueled TCA cycle. Mitochondrial respiration might participate in ROS production, thereby leading to a potential positive feedback loop in high-OXPHOS HGSOCs. High-OXPHOS HGSOCs exhibit an enhanced sensitivity to conventional therapies, an effect mediated at least in part through the ROS-PML axis described here. ROS, reactive oxygen species; NB, nuclear bodies; Ac, acetylated lysine; ETC, electron transport chain; TF, transcription factor; CI, complex I; CII, complex II; CIII, complex III; CIV, complex IV; CV, complex V; TCA, tricarboxylic acid; NADH, H^+^, nicotinamide adenine dinucleotide reduced form; FADH2, flavin adenine dinucleotide reduced form; OXPHOS, oxidative phosphorylation. Figure360: An Author Presentation of Figure 6

Consistent with results on PDX and patients, high-OXPHOS OCCLs exhibited higher chemosensitivity to taxane and platinum salts than low-OXPHOS cells (Figure 6A). Here again, this effect was not linked to HR status, as both high- and low-OXPHOS OCCLs included high-LST profile (STAR Methods). Notably, PML and PGC-1α silencing significantly reduced the chemosensitivity of high-OXPHOS OCCLs (Figures 6B and 6C). Moreover, PML silencing in mouse models also significantly reduced chemosensitivity *in vivo* (Figures 6D and 6E), suggesting that PML is essential not only to promote high-OXPHOS status (Figure 4), but also to modulate chemosensitivity. We next sought to define how high-OXPHOS status promotes chemosensitivity. We hypothesized that the chronic oxidative stress detected in high-OXPHOS tumors and OCCLs at basal state (Figure 3) could be involved in their increased chemosensitivity. We first confirmed that treatment of OCCLs with taxane and platinum salts significantly increased ROS content (Figure 6F), in particular in high-OXPHOS cells (fold change = 1.6 in high-OXPHOS; 1.1 in low-OXPHOS). In addition, although it was not possible to measure ROS content in tumors, we analyzed by electron microscopy the mitochondria integrity in PDX before and after chemotherapy. While sections showed normal ultrastructure and inner and outer membrane integrity before treatment, chemotherapy had a dramatic effect on mitochondrial integrity in high-OXPHOS PDX, as they exhibited altered ultrastructure and cristae disorganization (Figure 6G). Moreover, consistent with the reduced chemosensitivity upon PML inactivation (Figure 6B), PML silencing also decreased ROS content, lipid peroxidation, and lysosomal Fe^2+^ levels (Figures 6H–6J), suggesting that ROS content in high-OXPHOS cells might be a key element in their chemosensitivity.

High-OXPHOS OCCLs are characterized by ROS increase, elevated lipid peroxidation, and disruption of iron homeostasis, all features affected by PML silencing. Increased lipid peroxidation and disruption of iron homeostasis, associated with elevated ROS content, characterize ferroptosis, an iron-dependent cell death program (Dixon et al., 2012, Mai et al., 2017). We thus hypothesized that ferroptosis could be involved in enhanced chemosensitivity of high-OXPHOS cells and investigated the impact of ironomycin, a potent derivative of the natural product salinomycin, known to promote death consistent with ferroptosis in breast cancer cells (Mai et al., 2017). Ironomycin exhibited a selective and more potent activity on high-OXPHOS OCCLs than low-OXPHOS cells (Figure 6K). Ironomycin significantly increased iron accumulation, ROS, and lipid peroxidation in high-OXPHOS OCCLs, while it had no significant impact on low-OXPHOS cells (Figures 6L–6N). Furthermore, PML silencing reduced the sensitivity of high-OXPHOS cells to ironomycin (Figure 6O), thereby showing that this sensitivity is linked to PML and OXPHOS status. These findings thus suggest that enhanced chemosensitivity of high-OXPHOS cells could rely on ROS accumulation, mitochondrial alterations, and potentially ferroptosis.

## Discussion

Here, we highlight an unsuspected metabolic heterogeneity in HGSOCs based on OXPHOS patterns and link it to PML-PGC-1α and chemosensitivity. High-OXPHOS tumors are characterized by increased expression of ETC components and enhanced mitochondrial respiration. High-OXPHOS HGSOCs undergo a chronic oxidative stress that increases PML-NBs, which in turn stimulate PGC-1α transcriptional activity and expression of mitochondrial respiration genes. Finally, by studying pre-clinical models and cohorts of patients, we show that high-OXPHOS HGSOCs are highly sensitive to conventional chemotherapies and that chronic oxidative stress and PML play key roles in this chemosensitivity (Figure 6P).

Genomic and transcriptomic analyses have previously identified several molecular entities in HGSOCs (Tothill et al., 2008, Cancer Genome Atlas Research Network, 2011, Mateescu et al., 2011, Bentink et al., 2012, Verhaak et al., 2013, Konecny et al., 2014, Gruosso et al., 2015, Batista et al., 2016). Our proteomics and metabolomics studies refine these classifications and highlight metabolic entities within HGSOCs. Previous comparative analyses between cancers and normal tissues demonstrated a glycolytic switch toward the Warburg effect in ovarian cancers (Fong et al., 2011). These metabolic changes helped identify specific biomarkers, including phospholipids and acylcarnitine, which accumulate at abnormal levels in the plasma of ovarian cancer patients (Sutphen et al., 2004, Odunsi et al., 2005, Ke et al., 2015, Xie et al., 2017). In addition, low serum phospholipids were correlated with worse prognosis (Bachmayr-Heyda et al., 2017), as we observed for low-OXPHOS patients. Moreover, metabolomic profiling of serum and tumor tissue from HGSOC patients revealed hydroxybutyric acid metabolites as prognostic biomarkers associated with tumor burden and patient survival (Hilvo et al., 2016). Our observations provide an additive demonstration of heterogeneity in the carbon sources and catabolic pathways used by HGSOCs. Indeed, we demonstrated that high-OXPHOS ovarian cancer cells use mainly glutamine and fatty acids, as also recently described in other tumors (Caro et al., 2012, Vazquez et al., 2013, Camarda et al., 2016, Hensley et al., 2016, Farge et al., 2017). Moreover, we identify here an ROS-dependent PML-PGC-1α axis in defining the high-OXPHOS status in HGSOCs.

The origin of metabolic heterogeneity was shown to be highly dependent on the cancer type (Obre and Rossignol, 2015, Gentric et al., 2017). Genomic amplification of metabolic genes forms a core part of metabolic reprogramming in various cancers (Possemato et al., 2011, Haider et al., 2016, Iorio et al., 2016). In contrast, we observed similar amplification patterns between low- and high-OXPHOS HGSOCs, consistent with a recent report showing that CNAs in HGSOCs do not affect metabolic functions (Zhang et al., 2016). Here, we show that high-OXPHOS HGSOCs exhibit several hallmarks of chronic oxidative stress and that PML is involved in promoting the OXPHOS status and its related chemosensitivity. PML is a well-known tumor suppressor in leukemia (Gurrieri et al., 2004, de Thé et al., 2017). However, when detected in tumors, PML acts as a potent pro-apoptotic factor through activation of TP53 or Rb/E2F pathway (Vernier et al., 2011, Ablain et al., 2014, Niwa-Kawakita et al., 2017). Numerous studies have shown that cells and mice lacking PML are resistant to pro-apoptotic and pro-senescent stimuli (de Thé et al., 2017). These observations are consistent with the enhanced chemoresistance we observed *in vitro* and *in vivo* upon PML silencing in high-OXPHOS ovarian cancers. PML expression is associated with inactivation of *TP53*, the most highly mutated gene in HGSOCs (Cancer Genome Atlas Research Network, 2011). While a metabolic function of PML was proposed in breast cancer cells (Carracedo et al., 2012, Martín-Martín et al., 2016), it was never explored in ovarian cancers. PML regulates metabolism by modulating PGC-1α activation, a key regulator of mitochondrial functions in physiology and in cancer metabolism (Tan et al., 2016). Importantly, we demonstrate here that PML and PGC-1α are both necessary for high-OXPHOS features. Reciprocally, PGC-1α-PPAR-mediated mitochondrial biogenesis in low-OXPHOS cells is sufficient to increase high-OXPHOS characteristics, thereby suggesting that the PML-PGC-1α axis may act as one of the switches between high- and low-OXPHOS states, by regulating transcription of mitochondrial genes. Mechanistically, localization of PGC-1α into subnuclear structures allows its interaction with transcriptional cofactors and coregulators. It is thus plausible that PML-NBs could constitute an interface whereby PGC-1α interacts with transcriptional components and where its acetylation is dynamically controlled toward activation.

The association between OXPHOS status and chemosensitivity represents a promising therapeutic window, potentially for ROS-producing agents (Gentric et al., 2017, Saed et al., 2017) and ferroptosis activators. Inhibition of mitochondrial respiration sensitizes various cancer cells to conventional therapies (Roesch et al., 2013, Viale et al., 2014, Farge et al., 2017). It was shown that chemotherapy can promote selection and expansion of high-OXPHOS cancer stem cells (Liu et al., 2013, Vazquez et al., 2013, Vellinga et al., 2015, Farge et al., 2017). These chemoresistant dormant cancer cells exhibit low levels of ROS associated with slow-cycling activity and enhanced antioxidant detoxification capacity (Caro et al., 2012, Roesch et al., 2013, Vazquez et al., 2013, Sancho et al., 2015), in sharp contrast to high-OXPHOS ovarian cancer cells. Indeed, we show that high-OXPHOS ovarian cancer cells and tumor samples exhibit features of chronic oxidative stress. These hallmarks of oxidative stress (ROS content, lipid peroxides, and lysosomal Fe^2+^) are all affected by PML silencing and may explain, at least in part, the enhanced chemosensitivity of high-OXPHOS cells to taxane and platinum salts, potentially through ferroptosis (Mai et al., 2017). In that sense, we found that inhibition of mitochondrial complex I by metformin increases mitochondrial ROS content and cell death of high-OXPHOS cells, while it has no impact on low-OXPHOS cells. In addition to drugs promoting ROS increase, targeting metabolic properties of high-OXPHOS cells, by combining glutaminolysis or fatty acid inhibitors with chemotherapeutic drugs, may be a promising strategy to increase cell death and overcome drug resistance, as proposed in Obre and Rossignol (2015) and Gentric et al. (2017). Overall, our findings provide functional and molecular evidence of OXPHOS metabolic heterogeneity in HGSOCs and link them to an ROS-PML-PGC-1α axis and, critically, to chemotherapy response.

### Limitations of Study

We sought to evaluate the capacity of ovarian cancer cells to use fatty acids, as a source of carbon, by using exogenous fatty acids, such as palmitate. However, exogenous palmitate was highly toxic in these cells, thereby precluding measuring fatty acid incorporation or isotype profiling, as we did by using exogenous glucose and glutamine.

## STAR★Methods

### Key Resources Table

**Table:** 

REAGENT or RESOURCE	SOURCE	IDENTIFIER
**Antibodies**		

anti-Total OXPHOS Human WB Antibody Cocktail (a mixture of NDUFB8 (ab110242), Complex II subunit 30kDa (ab14714), Complex III subunit Core 2 (ab14745), Complex IV subunit II (ab110258), and ATP synthase subunit alpha (ab14748))	ABCAM	Cat#ab110411; RRID: AB_10859122, AB_301432, AB_2213640, AB_10887758, AB_301447
anti-PML	SANTA CRUZ	Cat#SC-5621; RRID: AB_2166848
anti-PGC-1α	Cell Signaling	Cat#9441; RRID: AB_2166218
anti-Actin	Sigma Aldrich	Cat#A5441; RRID: AB_476744
anti-K-Acetylated	Cell Signaling	Cat#9441; RRID: AB_331805
Peroxidase-conjugated secondary antibody (Mouse)	Jackson ImmunoResearch Laboratories	Cat#115-035-003
Peroxidase-conjugated secondary antibody (Rabbit)	Jackson ImmunoResearch Laboratories	Cat#115-035-045
rabbit IgG antibody	ABCAM	Cat#171870
goat polyclonal secondary antibody to rabbit IgG Alexa Fluor 488	ABCAM	Cat#ab150077

**Bacterial and Virus Strains**		

PLKO.1-puro derived vectors	Sigma Aldrich	Cat#SHC001
PLKO.1-puro derived vectors	Sigma Aldrich	Cat#TRCN0000003866
PLKO.1-puro derived vectors	Sigma Aldrich	Cat#TRCN0000003868

**Biological Samples**		

Ovarian frozen tumors	Institut Curie Hospital group	N/A
FFPE Ovarian sections	Institut Curie Hospital group	N/A
Patient-derived xenografts (PDX)	Institut Curie	N/A

**Chemicals, Peptides, and Recombinant Proteins**		

Target retrieval solution citrate pH 6	Dako	Cat#S2369
DharmaFECT	Dharmacon	Cat#T-2001-02
Lipofectamine 2000	Invitrogen	Cat#11668
Complete EDTA-free protease inhibitor cocktail	Roche	Cat#1836170
EDTA-free protease inhibitor cocktail tablet	Roche	Cat#1836170
NH_4_HCO_3_	Sigma Aldrich	Cat#T6567
Western Lightning Plus	PerkinElmer	Cat#NEL103E001EA
SyproRuby protein gel stain	Life Technologie	Cat#S12000
DMEM	Thermo Fisher Scientific	Cat#11995
Sodium pyruvate	Thermo Fisher Scientific	Cat# 11360070
L-glutamine	Thermo Fisher Scientific	Cat# 25030081
D-(+) Glucose solution	Sigma Aldrich	Cat#G8644
^13^C-D-glucose	Cambridge Isotope Laboratories	Cat#CLM-1396
^13^C-L-glutamine	Cambridge Isotope Laboratories	Cat# CLM-1822
Laemmli Sample buffer (2X)	Biorad	Cat#161-0737
4–12% polyacrylamide gel	Invitrogen	Cat#NP0323BOX - WG1403A
0.45 μm nitrocellulose transfer membrane	Sigma Aldrich	Cat#Z741975
Glutaraldehyde	Sigma Aldrich	Cat#G5882
Paraformaldehyde	Sigma Aldrich	Cat#P6148
Osmium tetroxide	Electron Microscopy Science	Cat#19100
Potassium ferrocyanure	Electron Microscopy Science	Cat#25154
MitoTracker Deep Red FM	Invitrogen	Cat#M22426
MitoTracker Red CMXRos	Invitrogen	Cat#M7512
tetramethylrhodamine, methyl ester	Thermo Fisher	Cat#T668
Seahorse XF Base Medium base pH 7.4	Agilent Technologies	Cat#103334-100
Seahorse calibration solution	Agilent Technologies	Cat#100840-000
Etomoxir	Sigma Aldrich	Cat#E1905
CB-839	Selleckchem	Cat#S7655
N-acetyl-L-Cystein	Sigma Aldrich	Cat#A7250
Rosiglitazone	Sigma Aldrich	Cat##R2408
Methanol	Sigma Aldrich	Cat#1060181000
Acetonitrile	Sigma Aldrich	Cat#1000291000
Formic Acid	Sigma Aldrich	Cat#5330020050
Puromycin	GIBCO	Cat#A11138-03
Agarose	Sigma Aldrich	Cat#A2576
Iodonitrotetrazolium chloride	Sigma Aldrich	Cat#I10406
DAPI	Invitrogen	Cat#D1306
Carboplatin	ACCORD	N/A
Paclitaxel	KABI	N/A
CellRox Reagent	Life Technologies	Cat#C10422
Lysosensor probe	Life Technologies	Cat#L7535
Bodipy C11 Reagent	Life Technologies	Cat# D3861
Metformin	Sigma Aldrich	Cat#317240
Resazurin reagent	Sigma Aldrich	Cat#R7017
Power SYBR Green PCR Master Mix	Applied Biosystems	Cat#4367659
RhoN_OX_M	Niwa-Kawakita et al., 2017	N/A
Ironomycin	Mai et al., 2017	N/A

**Critical Commercial Assays**		

BCA Protein Assay kit	Roche	Cat#1836170
Short Tandem Repeat (STR) DNA profiling	Promega	Cat# B9510
Seahorse XF Cell Mito Stress Test Kit	Agilent Technologies	Cat#103015-100
XF Glycolysis Stress Test Kit	Agilent Technologies	Cat#103020-100
Dynabeads Antibody Coupling Kit	Life Technologies	Cat#14311D
miRNEasy kit	QIAGEN	Cat#217004
iScript Reverse Transcription Kit	Bio-Rad	Cat#1708840

**Deposited Data**		

Original and analyzed data	This paper	https://doi.org/10.17632/fstsb2xfsf.1
Trancriptomic data from Curie Cohort	Mateescu et al., 2011	GEO: GSE26193

**Experimental Models: Cell Lines**		

SKOV3	ATCC	Cat# HTB-77
OV90	ATCC	Cat# CRL-11732
CAOV3	ATCC	Cat# HTB-75
OV7	Public Health England	Cat# 96020764
COV504	Public Health England	Cat# 07071902
OV56	Public Health England	Cat# 96020759
IGROV1	D. Lallemand and J.S. Brugge Lab	N/A
OVCAR8	R. Spizzo Lab	N/A
OC314	R. Spizzo Lab	N/A
KURAMOCHI	R. Spizzo Lab	N/A
OVSAHO	R. Spizzo Lab	N/A
OVCAR4	R. Spizzo Lab	N/A
FUOV1	R. Spizzo Lab	N/A
COV318	R. Spizzo Lab	N/A

**Experimental Models: Organisms/Strains**		

Female Swiss nude (6 week old)	Charles River	Cat# Crl:NU(Ico)-Foxn1nu

**Oligonucleotides**		

non-targeting siRNA	Dharmacon	Cat#D-001810-02
PML-targeting siRNA	Dharmacon	Cat#J-019734-06
PML-targeting siRNA	Dharmacon	Cat#J-019734-07
PGC-1α –targeting siRNA	Dharmacon	Cat# J-005111-05
PGC-1α –targeting siRNA	Dharmacon	Cat#J-005111-07
*PML-F*: 5′– GTGAAGGCCCAGGTTCAG –3′	Eurofins	N/A
*PML-R*: 3′– CCTCAGACTCCATCTTGATGAC –5′	Eurofins	N/A
*NDUFB8-F*: 5′– CTCCTTGTTGGGCTTATCACA –3′	Eurofins	N/A
*NDUFB8-R*: 3′– GCCCACTCTAGAGGAGCTGA –5′	Eurofins	N/A
*SDHB-F*: 5′– AAGCATCCAATACCATGGGG –3′	Eurofins	N/A
*SDHB-R*: 3′–TCTATCGATGGGACCCAGAC –5′	Eurofins	N/A
*UQCRC2-F*: 5′–GTTTGTTCATTAAAGCAGGCAGTAG –3′	Eurofins	N/A
*UQCRC2-R*: 3′– TGCTTCAATTCCACGGGTTATC –5′	Eurofins	N/A
*MTCO2-F*: 5′– TCATTTTCCTTATCTGCTTCC –3′	Eurofins	N/A
*MTCO2-F*R: 3′– ACGGTTTCTATTTCCTGAGC –5′	Eurofins	N/A
*COX4I1-F*: 5′– ATGTCAAGCACCTGTCTGC –3′	Eurofins	N/A
*COX4I1-R*: 3′– CCCTGTTCATCTCAGCAAA –5′	Eurofins	N/A
*ATP5A1-F*: 5′– ACTGGGCGTGTCTTAAGTATTG –3	Eurofins	N/A
*ATP5A1-R:* 3′– ACCAAGGGCATCAACTACAC –5′	Eurofins	N/A
*PPARGC1A-F*: 5′– CAGAGAACAGAAACAGCAGCA –3′	Eurofins	N/A
*PPARGC1A-R*: 3′– TGGGGTCAGAGGAAGAGATAAA –5′	Eurofins	N/A
*CYCLOPHILIN-B-F*: 5′– AGGCCGGGTGATCTTTGGTCT –3′	Eurofins	N/A
*CYCLOPHILIN-B-R*: 3′– CCCTGGTGAAGTCTCCGCCCT –5′	Eurofins	N/A

**Software and Algorithms**		

SEQUEST - Proteome Discoverer 1.4	Thermo Fisher Scientific	N/A
Metaboanalyst software	(http://www.metaboanalyst.ca)	N/A
cBioportal	https://portal.gdc.cancer.gov	N/A
iTEM software	Soft Imaging System	N/A
ImageJ	https://imagej.nih.gov/ij/, 1997-B014	N/A
GraphPad Prism software	https://www.graphpad.com	N/A
R versions 3.3.2 and 3.4.0	https://cran.r-project.org	N/A
REVIGO	http://revigo.irb.hr	N/A
FlowJo version 10.0.7	https://www.flowjo.com/solutions/flowjo	N/A
DAVID	https://david.ncifcrf.gov	N/A
Consensus clustering	Monti et al., 2003	N/A

**Other**		

24-well cell culture insert	Costar	Cat#3422

### Contact for Reagent and Resource Sharing

Further information and requests for resources should be directed to and will be fulfilled by the Lead Contact, Fatima Mechta-Grigoriou (fatima.mechta-grigoriou@curie.fr).

### Experimental Model and Subject Details

#### Cohorts of HGSOC Patients

Cohorts of ovarian cancer patients from Institut Curie (a total of 127 HGSOC patients; 45 samples used for metabolomics analysis, 127 for proteomic analysis and 71 for IHC) and TCGA (169 patients) have been previously described in Mateescu et al., 2011, Gruosso et al., 2015, and Batista et al. (2016), and Cancer Genome Atlas Research Network (2011) and Zhang et al. (2016), respectively. Main clinical features of these cohorts are summarized in Table S1 and its corresponding legend. The project developed here is based on surgical tumor tissues, from the Institut Curie Hospital Group, available after histo-pathological analyses and not needed for diagnosis. There is no interference with the clinical practice. Analysis of tumor samples was performed according to the relevant national law providing protection to people taking part in the biomedical research. Their referring oncologist informed all patients included in our study that their biological samples could be used for research purposes and patients signed an informed consent of non-opposition. In case of patient refusal, expressed either orally or written, residual tumor samples were excluded from our study. The Institutional Review Board and Ethics committee of the Institut Curie Hospital Group approved all analyses realized in this study, as well as the National Commission for Data Processing and Liberties (N° approval: 1674356 delivered on March 30, 2013).

#### Xenograft Models

All protocols involving mice and animal housing were in accordance with institutional guidelines as proposed by the French Ethics Committee and have been approved (agreement number: CEEA-IC #115: 2013-06). HGSOC models were established at the Institut Curie with patient consent according to the relevant national law on the protection for people participating in biomedical research. Main features of these models are summarized in Table S1.

*HRD-HRP status (for detailed on the method, see* Genomic Analysis in HGSOC*):* OV14, OV21, OV16, OV26 exhibit HRP profiles and OV25, OV38, OV10 exhibit HRD profiles

#### Human Ovarian Cancer Cell Lines

We used human epithelial ovarian cancer cell lines (OCCL) SKOV3, OV90, CAOV3 (from American Type Culture Collection, ATCC), OV7, COV504 and OV56 (from Public Health England), IGROV1 (a kind gift from D. Lallemand and J.S. Brugge) and OVCAR8, OC314, KURAMOCHI, OVSAHO, OVCAR4, FUOV1, COV318 (kind gifts from R. Spizzo). Each cell line identity was verified by Short Tandem Repeat (STR) DNA profiling (Promega, # B9510). Cells were grown in DMEM (GIBCO, Thermo Fisher Scientific #11995) with glucose (4.5 g / L), 4 mM L-glutamine (Thermo Fisher Scientific # 25030081), 1 mM sodium pyruvate (Thermo Fisher Scientific # 11360070) supplemented with 10% fetal bovine serum (FBS, BioSera #FB-1003/500), penicillin (100 U / ml) and streptomycin (100 μg / ml; Thermo Fisher Scientific # 15140122) in a humidified atmosphere of 5% (v/v) CO_2_ in air at 37°C. Cells were maintained for a maximum of 25 passages and testing confirmed the absence of mycoplasma contamination.

*HRD/HRP status (for detailed on the method, see* Genomic Analysis in HGSOC*):* IGROV-1, SKOV3, COV504, KURAMOCHI, OVSAHO, FUOV1, OC314, OV90 and COV318 cell lines exhibit HRP profiles. OVCAR4 and OVCAR8 exhibit HRD profiles. Data is unavailable for OV7, OV56 and CAOV3 cell lines.

### Method Details

#### Proteomic and Western Blot Analysis from HGSOC

##### Protein Extracts

Proteins were extracted from 127 frozen HGSOC enriched in epithelial cancer cells (on average, 73% of the tumor was composed of epithelial cells, with 50% being the minimum) were extracted using boiling lysis buffer (50 mM Tris pH 6.8, 2% SDS, 5% glycerol, 2 mM DTT, 2.5 mM EDTA, 2.5 mM EGTA, 4 mM Na3VO4 and 20 mM NaF) supplemented with 2x Halt Phosphatase inhibitor (Perbio #78420) and complete EDTA-free protease inhibitor cocktail tablet (Roche #1836170). The protein extract was snap frozen in liquid nitrogen and stored at −80°C. Protein concentration was evaluated using the BCA Protein Assay kit – Reducing Agent Compatible according to the manufacturer’s instructions (Thermo Fisher Scientific # 23250).

##### Label-free Quantitative Proteomics

Proteins were extracted from 127 frozen HGSOC, as described above, and digested with trypsin at 10 ng / μl in 50 mM NH_4_HCO_3_ (Sigma Aldrich #T6567). Two independent runs of analysis were performed: the first with 60 HGSOC protein samples and the second with 67 HGSOC protein samples. Peptide mixture was analyzed on a Ultimate 3000 nanoLC system (Dionex, Amsterdam, the Netherlands) coupled to an Electrospray Q-Exactive quadrupole Orbitrap benchtop mass spectrometer (Thermo Fisher Scientific). 10 μl of peptide digests were loaded onto a 300-μm-inner diameter x 5-mm C18 PepMapTM trap column (LC Packings) at a flow rate of 30 μl / min. Peptides were eluted from the trap column onto an analytical 75-mm id x 25-cm C18 Pep-Map column (LC Packings) with a 4%–40% linear gradient of solvent B in 108 min (min) (solvent A was 0.1% formic acid in 5% Thermo Scientific Acetonitrile (CAN) and solvent B was 0.1% formic acid in 80% ACN). The separation flow rate was set at 300 nL / min. The mass spectrometer operated in positive ion mode at a 1.8-kV needle voltage. Data were acquired using Xcalibur 2.2 software in a data-dependent mode. Mass spectrometry scans (m/z 350-1600) were recorded at a resolution of R = 70 000 (@ m/z 200) and an automatic gain control (AGC) target of 3 × 10^6^ ions collected within 100 ms. Dynamic exclusion was set to 30 s and the top 12 ions were selected from fragmentation in higher energy collisional dissociation (HCD) mode. MS/MS scans with a target value of 1 × 10^5^ ions were collected with a maximum fill time of 100 ms and a resolution of R = 17500. Additionally, only +2 and +3 charged ions were selected for fragmentation. Others settings were as follows: no sheath nor auxiliary gas flow, heated capillary temperature, 250°C; normalized HCD collision energy of 27%, and an isolation width of 2 m/z.

##### nLC-MS/MS Analysis

Data were searched by SEQUEST through Proteome Discoverer 1.4 (Thermo Fisher Scientific) against a subset of the 2016.01 version of UniProt database restricted to *Homo sapiens* Reference Proteome Set (70,671 entries). Spectra from peptides higher than 5000 Dalton (Da) or lower than 350 Da were rejected. The search parameters were as follows: mass accuracy of the monoisotopic peptide precursor and peptide fragments were set to 10 ppm and 0.02 Da, respectively. Only b- and y-ions were considered for mass calculation. Peptide validation was performed using the Percolator algorithm and only “high confidence” peptides were retained corresponding to 1% false positive rate at peptide level. Raw LC-MS/MS data were imported in Progenesis QI for Proteomics 2.0 (Nonlinear Dynamics Ltd, Newcastle, U.K). Data processing included the following steps: (i) Features detection, (ii) Features alignment across all samples, (iii) Volume integration for 2-6 charge-state ions, (iv) Normalization on total protein abundance, (v) Import of sequence information, (vi) Calculation of protein abundance (sum of the volume of corresponding peptides). Only non-conflicting features and unique peptides were considered for calculation at the protein level. Quantitative data were considered for peptides with a signal > 10,000 for at least 5% of samples. A minimum of 2 unique peptides was required to identify a given protein within the full dataset. Data was standardized per run. All detected peptides in low- and high-OXPHOS HGSOC are listed in Table S4.

##### Proteomic Data from the TCGA Cohort

Proteomic data from the TCGA cohort was extracted from Table mmc3 in Zhang et al. (2016), with the same procedure for final protein quantification, as described in the paper. Identification of low- and high-OXPHOS tumors from Curie and TCGA proteomic data: the consensus clustering method described in Monti et al. (2003) was applied on Curie and TCGA cohorts using a list of 27 ETC proteins in order to identify the optimal number of tumor subgroups. The following parameters were used: clustering method based on *k-means*, 1000 iterations, 80% of sample resampling. In both cases, classification in 2 metabolic subgroups was identified as the most robust.

##### Western Blots

20 μg proteins extracted from HGSOC were loaded onto homemade 12% polyacrylamide gels. After electrophoresis, the proteins were transferred to a 0.45 μM PVDF transfer membrane (Immobilon-P, Millipore, #IPVH 00010) and blotted overnight at 4°C with the appropriate primary antibodies: a cocktail of 5 ETC proteins: ATP5A: ATP Synthase, H^+^ Transporting, Mitochondrial F1 Complex, Alpha Subunit, UQCR2: Ubiquinol-Cytochrome C Reductase Core Protein II, SDHB: Succinate Dehydrogenase Complex Iron Sulfur Subunit B, COXII: Mitochondrially Encoded Cytochrome C Oxidase II, NDUFB8: NADH:Ubiquinone Oxidoreductase Subunit B8 (1:2000; ABCAM #ab110411) and Actin (1:10,000; Sigma #A5441). Specific binding of antibodies was detected using appropriate peroxidase-conjugated secondary antibodies (Jackson ImmunoResearch Laboratories #115-035-003), and was visualized by enhanced chemiluminescence detection (Western Lightning Plus-ECL, PerkinElmer #NEL103E001EA). Densitometry analyses of immunoblots were performed using ImageJ software.

#### Metabolomic Analysis of HGSOC Samples

Metabolomic analysis of 45 HGSOC from the Curie cohort was performed by Metabolon. (Morrisville, USA). Briefly, samples enriched in at least 50% of epithelial cancer cells were extracted and prepared for analysis using Metabolon’s standard solvent extraction method. The extracted samples were split into equal parts for analysis on the gas chromatography mass spectrometry (GC/MS) and liquid chromatography tandem-mass spectrometry (LC/MS/MS) platforms to allow the detection of 374 biochemicals. Quality controls (QC) were performed using internal standards and Metabolon QC samples. All detected metabolites in low- and high-OXPHOS HGSOC are listed in Table S5.

For sparse partial least square discriminant analysis (sPLS-DA), data were filtered by interquartile range, normalized to the median and Log-transformed. 200 metabolites were used for each component. sPLS-DA was performed by using Metaboanalyst software (http://www.metaboanalyst.ca).

#### Genomic Analysis in HGSOC

##### Homologous Recombination (HR) Status - LST Signature

CytoscanVR HD SNP-array (Affymetrix) data were processed using the GAP methodology to obtain absolute copy number profiles, as in Popova et al. (2012). DNA index was calculated as the average copy number. Based on the DNA index, tumor ploidy was set as near-diploid (DNA index < 1.3) or near-tetraploid (DNA index > = 1.3). Genomic HRD was detected based on the number of LST (Popova et al., 2012). Briefly, LST was defined as a chromosomal breakpoint (change in copy number or major allele counts) between adjacent regions of at least 10 Megabases. The number of LST was calculated after smoothing and filtering out copy number variant regions < 3 Megabases in size. Tumors were segregated into near-diploid or near-tetraploid subgroups. Based on two ploidy-specific cut-offs (15 and 20 LST per genome in near-diploid and near-tetraploid tumors respectively) tumors were classified as HRD (high-LST, equal to or above the cut-off) or HRP (low-LST, below the cut-off).

##### CNA and Mutations

Genomic data from the TCGA were extracted from the NIH Genomic Data Common (GDC) data portal (https://portal.gdc.cancer.gov). For DNA copy number alteration, data were available for 88 low-OXPHOS and 79 high-OXPHOS HGSOC. Mutation status was extracted for 46 low-OXPHOS and 43 high-OXPHOS HGSOC. *BRCA1* and *RAD51C* promoter methylation status was defined in the TCGA cohort, as described in Manié et al. (2016).

#### Transcriptomic Analysis from HGSOC

Trancriptomic analysis from Curie Cohort was described in Mateescu et al. (2011). Data are freely accessible in the Gene Expression Omibus under the accession number GEO: GSE26193. For the TCGA cohort, freely available transcriptomic data (Cancer Genome Atlas Research Network, 2011) have been downloaded from the following portal: https://cancergenome.nih.gov/.

#### Protein Carbonylation Analysis from HGSOC

Tissue proteins were extracted from 40 HGSOC. Protein oxidation was measured by OxiProteomics (https://www.oxiproteomics.fr/). Extracted proteins were quantified by the Bradford method and split into equal amounts for analyses. Carbonylated proteins were labeled with specific functionalized fluorescent probes and samples were resolved by high-resolution electrophoresis separation. Total proteins were post-stained with SyproRuby protein gel stain (Life Technologies, #S12000). Image acquisition for carbonylated and total proteins was performed using the Ettan DIGE imager (GE Healthcare). Image processing and analysis was performed using ImageJ (Rasband, W.S., ImageJ, National Institutes of Health, USA, https://imagej.nih.gov/ij/, 1997-B014). Density histograms and lane profile plots were obtained from each sample, both for carbonylated and total proteins. Carbonylated protein signal was normalized by total protein signal for each sample in order to obtain the carbonylation score (Score = carbonylated protein / total protein).

#### Immunohistochemistry in HGSOC and OCCL

##### IHC and Validation of PML-Directed Antibody

71 samples from the Curie cohort were first selected by pathologists based on tumor grade, histological subtype and clinical features for considering only HGSOC. For IHC, sections of paraffin-embedded tissues (3 μm) were stained using a streptavidin-peroxidase protocol and the Lab Vision Autostainer 480 (Thermo Fisher Scientific), as described in Gruosso et al. (2015). Paraffin-embedded sections were incubated with specific antibodies recognizing PML (1:200; Santa Cruz #SC-5621) or rabbit IgG antibody (1:500; ABCAM #171870) in Phosphate Buffered Saline solution at pH 7.6 containing 0.05% Tween 20 for 1 hour, following unmasking in 1 x Citrate buffer, pH 6 (Dako #S2369) for 15 min at 95°C. For quantification, the whole section was considered and evaluated by two independent researchers. Histological scores (Hscore) of PML staining in epithelial cells were given as a function of the percentage of positive epithelial cells multiplied by the staining intensity (ranging from 0 to 3): Hscore = Intensity of staining x % of stained cells. The specificity of the PML antibody was verified using OV56 and OVCAR4 ovarian cancer cell lines in which PML has been or not silenced (see also PML and PGC-1α Silenced Cell Lines). In brief, 20 × 10^6^ cells were plated into 15 cm Petri dishes. 24h post plating, cells were washed with room temperature PBS, trypsinized and pelleted in PBS before fixation using alcohol, formalin and acetic acid (AFA) fixative followed by paraffin-embedding. Sections of AFA-fixed paraffin-embedded cells (3 μm) were stained using the protocol described above for human HGSOC samples.

##### PML Nuclear-Body (NB) Quantification

Quantification of PML-NB corresponds to the number of PML foci divided by the number of cells. Quantification was assessed in two steps by ImageJ software and further confirmed by visual inspection of images. (1) Evaluation of cell number, which was automatically estimated by applying a threshold filter (0.180) followed by a mask conversion, and then particles were analyzed (size > 80; circularity > 0.4). (2) PML-NB quantification: PML-NB were automatically detected by applying a threshold filter (0.150) followed by a mask conversion. Particles were analyzed (size = 1-25; circularity > 0.6). PML-NB were evaluated in 71 HGSOC, with an average of 12 (and a minimum of 8) slides analyzed per tumor.

#### Protein Extracts and Western Blots from OCCL

For *protein level analyses*, cell lines were plated into six-well plates (Corning #353046) in DMEM with 10% FBS to reach 80% confluence 48 hr after plating, depending on doubling time. Cells were washed with PBS and scraped with 2 x Laemmli Sample buffer (Biorad #161-0737) with 0.1 mM DTT. Samples were boiled for 5 min at 95°C followed by centrifugation at 13,000 rpm for 10 min at 4°C to eliminate cell debris. Protein samples were transferred into a fresh tube and the protein concentration was determined using the BCA Protein Assay kit – Reducing Agent Compatible according to the manufacturer’s instructions (Thermo Fisher Scientific # 23250).

For *western blot analysis*, 10 μg proteins were loaded onto precast 4%–12% polyacrylamide gels (Invitrogen #NP0323BOX - WG1403A). After electrophoresis, proteins were transferred to a 0.45 μm nitrocellulose transfer membrane (Sigma Aldrich # Z741975) and incubated overnight at 4 °C with the appropriate primary antibodies: a cocktail of 5 ETC proteins (listed above, Proteomic Data from the TCGA Cohort) (1:2000; ABCAM #ab110411), PML (1:2000; Santa Cruz #SC-5621), PGC-1α (1:500; Santa Cruz #sc-13067), K-Acetylated (1:2000; Cell Signaling #9441) and Actin (1:10,000; Sigma Aldrich #A5441). Specific binding of antibodies was detected using appropriate peroxidase-conjugated secondary antibodies (Jackson ImmunoResearch Laboratories #115-035-003 and 115-035-045) and visualized by enhanced chemiluminescence detection (Western Lightning Plus-ECL, PerkinElmer). Densitometric analyses of immunoblots were performed using ImageJ software. Actin was used as an internal control for protein loading and normalization.

#### Mitochondrial Content and Structure Analysis

##### Electron Microscopy on OCCL and PDX Tumor Samples

Cells were seeded onto 24-mm glass coverslips (VWR #631-0161), allowed to grow to 50%–60% confluency and were fixed in 2.5% glutaraldehyde (Sigma Aldrich # G5882) and 2% paraformaldehyde (Sigma Aldrich # P6148). Tumor samples from PDX were directly fixed under same conditions. All samples were post-fixed in 1% osmium tetroxide (Electron Microscopy Science # 19100) containing 1.5% potassium ferrocyanure (Electron Microscopy Science # 25154). Samples were embedded in EPON and ultrathin sections were contrasted with uranyl acetate and lead citrate. Electron micrographs were acquired on a Tecnai Spirit electron microscope (FEI, Eindhoven, the Netherlands) equipped with a 4k CCD camera (Quemesa, EMSIS GmbH, Münster, Germany). For image analysis and quantification, mitochondrial area was evaluated on randomly selected cells using iTEM software (Soft Imaging System, EMSIS GmbH, Germany). The mean area of each group was determined on 8 independent images, evaluating 2000 μm^2^ per cell line. Size of measured structures was expressed in μm^2^.

##### Mitochondrial Staining Using MitoTracker Probe

Cells were seeded onto six-well plates and grown up to 70% confluency. Mitochondrial content per cell line was estimated using MitoTracker Deep Red FM (Molecular Probes/Invitrogen #M22426). For assessment of mitochondrial membrane potential, cells were stained with MitoTracker Red CMXRos (Molecular Probes/Invitrogen #M7512) and tetramethylrhodamine, methyl ester (TMRM, Thermo Fisher #T668). Cells were stained with 250 nM MitoTracker Deep Red FM or 250 nM MitoTracker Red CMXRos or 100 nM TMRM for 30 min at 37°C. Cells were then washed with PBS solution, trypsinized, and resuspended in PBS solution containing 1% FBS for flow cytometric analysis. Flow cytometry data were acquired using an LSR FORTESSA analyzer (BD biosciences).

#### Seahorse Technology

Cells were seeded (4 replicates) in XFe96 Cell Culture Microplates (Seahorse, Bioscience #101085-004) at 80%–90% confluency in DMEM supplemented with 10% FCS ± 10 mM Glucose, ± 2 mM Glutamine, ± 1 mM Pyruvate. Cells were incubated for 24 hours at 37°C in 5% CO_2_ atmosphere. Before the experiment, the culture medium was removed from each well and replaced with 175 μL of serum-free unbuffered Seahorse XF Base Medium base pH 7.4 (Agilent Technologies #103334-100) pre-warmed at 37°C and supplemented with 10 mM glucose, 2 mM glutamine and 1 mM Pyruvate (for analysis of mitochondrial oxidative metabolism) or with 0 mM glucose (for analysis of glycolysis assessment). Cells were incubated in a CO_2_ free incubator at 37°C for 1 h. Cartridges equipped with oxygen- and pH-sensitive probes were preincubated with calibration solution (Agilent Technologies #100840-000) overnight at 37°C in an incubator without CO_2_. Prior to the rate measurements, the XF96 Analyzer (Seahorse biosciences, North Billerica, MA) automatically mixed the assay media in each well for 15 min to allow the oxygen partial pressure to reach equilibrium. Oxygen consumption rate (OCR) and extracellular acidification rate (ECAR) were evaluated in a time course before and after injection of the following compounds: OCR measurement (using Agilent Technologies #103015-100) (i) 1 μM Oligomycin; (ii) 0.5 μM FCCP [Carbonyl cyanide-4-(trifluoromethoxy)phenylhydrazone; (iii) 0.5 μM Antimycin A + Rotenone / ECAR measurement (using Agilent Technologies #103020-100): (i) 10 mM Glucose; (ii) 1 μM Oligomycin; (iii) 50 mM 2-deoxyglucose (2-DG). A volume of 25 μL of compound was added to each injection port, and 3 baseline measurements were taken prior to the addition of any compound. After a 3 min wait, 3 response measurements were taken after each addition. ECAR and OCR values were normalized to the total amount of protein per well. ECAR and OCR data points refer to the average rates during the measurement cycles and were reported as absolute rates (mpH / min for ECAR, pMoles / min for OCR). For experiment testing carbon source preference, cells were incubated overnight in DMEM ± 10 mM Glucose, ± 2 mM Glutamine. Basal ECAR/OCR measurement was performed the following day. For fatty acid oxidation or glutaminolysis inhibition experiments, cells were incubated overnight in DMEM ± 10 mM Glucose, ± 2 mM Glutamine. OCR was measured 30 min after Etomoxir treatment (40 μM, Sigma Aldrich #E1905) or 1 hour after CB-839 treatment (10 μM, Selleckchem #S7655), respectively. For modulating OCR experiment, cells were incubated 48 hours in N-acetyl-L-Cystein (NAC at 5mM, Sigma Aldrich #A7250) or Rosiglitazone (at 20 μM, Sigma Aldrich #R2408) before OCR measurement.

#### Isotope Profiling in OCCL

##### Cultivation, Sampling and Metabolite Extraction

6 × 10^5^ IGROV1 and OC314 cells were seeded onto 30-mm glass coverslips and were incubated the day after in no glucose, no glutamine and no pyruvate DMEM complemented with 10 mM ^13^C-D-glucose (Cambridge Isotope Laboratories #CLM-1396) + 2 mM of glutamine or 2 mM ^13^C-L-glutamine (Cambridge Isotope Laboratories #CLM-1822) + 10 mM of glucose for 24 hours. Intracellular metabolites were extracted at −20°C with 8 mL of acetonitrile/methanol/water+0.1% of formic acid (2:2:1) and cells were scraped from the cover glasses. The solution was sonicated for 30 s and incubated for 15 min on ice for the metabolite extraction. Subsequently, the sample was frozen with liquid nitrogen, freeze-dried and finally re-extracted with an aqueous solution before mixing with the appropriate solvent for LC-MS analysis.

##### LC-MS Analysis

Analysis of intracellular amino acids was performed by liquid chromatography (HPLC U3000, Dionex, Sunnyvale, CA, USA) coupled with a LTQ Orbitrap Velos mass spectrometer (Thermo Fisher Scientific, Waltham, MA, USA) equipped with a heated ESI probe. MS analyses were performed in the positive FTMS mode at a resolution of 60,000 (at m/z 400). Analysis of intracellular central metabolites was performed by high performance anion exchange chromatography (Dionex ICS 5000+ system, Sunnyvale, USA) coupled with a LTQ Orbitrap Velos mass spectrometer (Thermo Fisher Scientific, Waltham, MA, USA) equipped with a heated ESI probe. Samples were analyzed in the negative FTMS mode at a resolution of 60,000 (at m/z 400). Isotopic cluster of each amino acids and central metabolites was determined by extracting and integrating the exact mass of all 13C-isotopologues with Tracefinder software (Thermo Fisher Scientific). Isotopic cluster of each amino acids and central metabolites was determined by extracting and integrating the exact mass of all 13C-isotopologues with Tracefinder software (Thermo Fisher Scientific). The correction was performed with IsoCor adapted for higt resolution mass spectrometry. Carbon isotopolog distributions were expressed relative to the sum of all analyzed isotopologs.

##### NMR Sample Preparation

Medium supernatants were mixed with D2O containing the internal standard TSP-d4 (Trimethylsylilpropionic acid d4) at 0.5 mM with 0.25 M DCl 0.025 M before analysis.

#### PML and PGC-1α Silenced Cell Lines

For generation of PML-silenced stable cell lines from CAOV3, OC314 and OVCAR4 OCCL, PLKO.1-derived vectors with two different shRNAs targeting human PML (TRCN0000003866 and TRCN0000003868 for shPML#1 and shPML#2, respectively), or expressing a scrambled shRNA (shCTRL #SHC001), were purchased from Sigma-Aldrich. Viruses were produced by co-transfection (with Lipofectamine 2000, Invitrogen #11668) of 293T cells with the vector plasmid, a vesicular stomatitis virus envelope expression plasmid (Vsvg) and a second-generation packaging plasmid (pPax2). Purified viral particles were used at multiplicity of infection 5 to infect CAOV3, OC314 and OVCAR4 OCCL overnight. Infected cells were selected with puromycin (1 μg ml^−1^) (GIBCO #A11138-03) for 1 week, before experimental use. Stable cell lines were propagated in DMEM (GIBCO, Thermo Fisher Scientific #11995) with glucose (4.5 g / l), 4 mM L-glutamine (Thermo Fisher Scientific # 25030081), 1 mM sodium pyruvate (Thermo Fisher Scientific # 11360070) supplemented with 10% fetal bovine serum (FBS, BioSera #FB-1003/500), penicillin (100 U / ml) and streptomycin (100 μg / ml; Thermo Fisher Scientific # 15140122) and 1 μg ml^−1^ of puromycin (GIBCO# A11138-03).

For the short interfering RNA (siRNA) experiment, 2-3 × 10^5^ cells were plated in six-well plates to reach 80% confluency after 3 days, depending on doubling time. After 24 hr, cells were transfected with 20 nM of non-targeting siRNA (siCtrl, Dharmacon #D-001810-02) or PML-targeting siRNA (Dharmacon siPML#1: #J-019734-06; siPML#2: #J-019734-07; siPML#pool, a mix of 4 individual siPML #J-019734-05 / 06 / 07 / 08) or PGC-1α-targeting siRNA (Dharmacon siPGC-1α#1: #J-005111-05; siPGC-1α#2: #J-005111-07) using 4 μL of DharmaFECT 1 transfection reagent in 2 mL final volume according to the manufacturer’s instructions (Dharmacon #T-2001-02).

#### Growth, Migration and Anchorage Independent Growth

##### Growth Kinetics

Cells were seeded at 2 × 10^4^ cells per well in 24-well plates (Corning #353047) and at indicated time points counted using Vi-Cell analyzer (Beckman Coulter). The number of living cells was measured by trypan blue exclusion.

##### Migration Assays

24-well cell culture insert (Costar # 3422, 8 μm pore size) were used for migration assay. After 24 hr serum starvation, 5 × 10^4^ cells were plated to the upper side of the Transwell device, in triplicates, in 100 μL of serum-free medium, whereas the lower well contained 600 μl of regular 10% FBS culture medium in order to create an FBS gradient. We ended the experiment after O.N. incubation. At the end of the experiment, the remaining cells in the upper side of the Transwell device were removed. Migrating cells at the bottom side of the Transwell device were fixed and stained with crystal violet for 20 min and then counted in 5 different representative fields (x 10 objective, Zeiss Axioplan microscope, AxioCamERc 5 s).

##### Soft Agar Assays for Anchorage-Independent Growth

4 × 10^4^ cells were passed 4-5 times through a 21G syringe, resuspended in complete medium with 0.3% agarose (Sigma Aldrich #A2576) and appropriate antibiotics and layered onto a 15 mL tubes (BD Biosciences, #352059) overlaid with medium without agarose. After two weeks, growth media was removed and viable colonies were stained with 2.5 mg / ml iodonitrotetrazolium chloride (Sigma Aldrich #I10406), scanned and finally quantified using the ImageJ software. All these experiments were done in at least 3 replicates.

#### PML Immunofluorescence

3 × 10^5^ cells were seeded onto glass coverslips placed inside a six-well plate. 48 hr later cells were fixed in 4% paraformaldehyde for 20 min, rinsed in PBS and blocked for 15 min in 3% BSA and 0,1% Triton. Cells were incubated with a specific antibody recognizing PML (1:500; Santa Cruz #SC-5621) for 45 min followed incubation with a goat polyclonal secondary antibody to rabbit IgG (Alexa Fluor 488, 1:1000, ABCAM #ab150077). Cells were stained with DAPI (2 μL / ml, Invitrogen #D1306) for nuclei detection. Slides were examined using an Upright Epifluorescence Microscope with Apotome (Zeiss), and images were acquired with identical exposure times and settings using a digital camera. Fluorescence image analysis was performed using the ImageJ software. For antioxidant impact on PML-NB, cells were treated with N-acetyl-L-Cystein (NAC at 5 mM, Sigma #A7250) for 48 hr and then processed as described above.

#### Features of Oxidative Stress

Briefly, cells were seeded onto six-well plates and grown up to 70% confluency, incubated directly with fluorescent probes for basal conditions. Then excess reagent was removed by washing the cells with PBS, trypsinized and resuspended in PBS solution containing 1% FBS for flow cytometric analysis. Flow cytometry data were acquired using an LSR FORTESSA analyzer (BD biosciences). For ROS quantification upon treatment, cells were treated for 24 hr with Carboplatin (at [5.10^−5^ M] ACCORD, 10 mg / ml) and Paclitaxel (at [10^−6^ M], KABI, 6 mg / ml) or Ironomycin (at 6 μM, synthesis is described in Mai et al., 2017), and then processed, as described above.

##### Cellular ROS

Cells were incubated with 2 μM CellRox Reagent (Life Technologies, #C10422) for 30 min at 37°C in the dark.

##### Lysosomal Fe^2+^ Content

Cells were incubated with 5 μM RhoM probes (RhoN_OX_-M lysosomal specificity for 60 min at 37°C in the dark. For normalization to lysosomal content that varies between cells, Lysosensor probe (1 μM, Life Technologies, #L7535) was used. The formula was applied: lysosomal Fe^2+^ = RhoM speMFI / Lysotracker speMFI.

##### Lipid Peroxide Product

Cells were incubated with 2 μM Bodipy C11 Reagent (Life Technologies # D3861) for 60 min at 37°C in the dark.

For Bodipy C11 IF, 3 × 10^5^ cells were seeded onto glass coverslips placed inside a six-well plate. 48 hr later cells were incubated with 2 μM Bodipy C11 Reagent for 60 min and then fixed in 4% paraformaldehyde for 20 min, rinsed in PBS. Slides were examined using an Upright Epifluorescence Microscope with Apotome (Zeiss) and images were acquired with identical exposure times and settings using a digital camera.

#### Immunoprecipitation

2 × 10^6^ CAOV3, OC314 and OVCAR4 OCCL were plated into 10 cm Petri dishes (Corning #353003). 24 hours later, cells were transiently silenced for PML (see PML and PGC-1α Silenced Cell Lines). 48 hr post transfection, cells were washed with cold PBS and scraped on ice. Cell suspensions were centrifuged at 13,000 rpm for 10 min at 4°C. Cell pellets were flash frozen in liquid nitrogen, resuspended in IP lysis buffer (50 mM HEPES pH 7.5, 150 mM NaCl, 1 mM EDTA, 1 mM EGTA, 10% glycerol, 1% Triton X-100, 25 mM NaF, 1 mM Na3VO4, 10 mM β-glycerophosphate, 5 mM sodium pyrophosphate, 0.5 μM PMSF) supplemented with EDTA-free protease inhibitor cocktail tablet (Roche #1836170) and incubated on ice for 20 min with vortexing every 5 min. Cell extracts were centrifuged at 13,000 rpm for 10 min at 4°C and supernatants were transferred into fresh tubes. The protein concentration was determined using the BCA Protein Assay kit – Reducing Agent Compatible according to the manufacturer’s instructions (Thermo Fisher Scientific # 23250). For immunoprecipitation, 300 μg of fresh protein extract were incubated overnight at 4°C with rotation, with 50 μL of PGC-1α (SantaCruz #sc-13067) coupled to magnetic beads (Dynabeads antibody coupling kit, Invitrogen #1143.11D) at 2 μg antibody per mg dynabeads. Beads were washed three times using IP lysis buffer. Lastly, 50 μL of samples buffer 2x (Biorad #1610737) were added on top of the beads and boiled for 5 min at 95°C. Western blot analysis of IP samples was performed as described above.

#### Cell Treatments and Cell Viability Assays

10^4^ cells were seeded per well in 96-well plates in DMEM medium with 10% FCS. Carboplatin (ACCORD, 10 mg / ml) and Paclitaxel (KABI, 6 mg / ml), or Ironomycin (in-house drug), or CB-839 (at 10 μM, Selleckchem #S7655)), or Metformin (at 0.01 M, Sigma Aldrich #317240) were added the next day at the appropriate concentration. Cell viability was assayed for IC_50_ determination at 48 hr for Carboplatin + Paclitaxel and at 72 hr for Ironomycin treatment or at 96 hr for the time course experiment by using the resazurin assay. To do so, 20 μL of resazurin reagent (0.05 mg / ml; Sigma Aldrich #R7017) was added to each well. Plates were incubated at 37°C for 2 hr and read in a Multi Detection plate reader (Fluostar, BMG Labtech).

#### qRT-PCR from Cell Lines

For gene expression analysis, total RNA isolation was performed using miRNEasy kit (QIAGEN, #217004) according to the manufacturer’s instructions. RNA concentrations were determined using a NanoDrop apparatus (NaNodrop Technologies). For each sample, 1 μg of total RNA was reverse transcribed using an iScript Reverse Transcription Kit (Bio-Rad #1708840). qRT-PCR was performed using Power SYBR Green PCR Master Mix (Applied Biosystems, #4367659) on a Chromo4 Real-Time PCR detection System (Bio-Rad) with primers at 300 nM final concentration. Primers (forward and reverse) used for quantitative (q)RT–PCR amplification were: *PML*: 5′– GTGAAGGCCCAGGTTCAG –3′; 3′– CCTCAGACTCCATCTTGATGAC –5′. *NDUFB8*: 5′– CTCCTTGTTGGGCTTATCACA –3′; 3′– GCCCACTCTAGAGGAGCTGA –5′. *SDHB*: 5′– AAGCATCCAATACCATGGGG –3′; 3′–TCTATCGATGGGACCCAGAC –5′. *UQCRC2*: 5′– GTTTGTTCATTAAAGCAGGCAGTAG –3′; 3′– TGCTTCAATTCCACGGGTTATC –5′. *MTCO2*: 5′– TCATTTTCCTTATCTGCTTCC –3′; 3′– ACGGTTTCTATTTCCTGAGC –5′. *COX4I1*: 5′– ATGTCAAGCACCTGTCTGC –3′; 3′– CCCTGTTCATCTCAGCAAA –5′. *ATP5A1*: 5′– ACTGGGCGTGTCTTAAGTATTG –3′; 3′– ACCAAGGGCATCAACTACAC –5′. *PPARGC1A*: 5′– CAGAGAACAGAAACAGCAGCA –3′; 3′– TGGGGTCAGAGGAAGAGATAAA –5′. *CYCLOPHILIN-B*: 5′– AGGCCGGGTGATCTTTGGTCT –3′; 3′– CCCTGGTGAAGTCTCCGCCCT –5′. Expression levels were normalized to *CYCLOPHILIN-B* and represented as fold change compared to the control (2ˆ(-ΔΔCt)). For evaluation of siRNA or drug impacts on gene expression, cells were incubated 48 hours with specific siRNA or with N-acetyl-L-Cystein (NAC at 5 mM, Sigma Aldrich #A7250) or Rosiglitazone (at 20 μM, Sigma Aldrich, #R2408) before RNA isolation.

#### Xenograft Experiment

##### Tumor Growth Analysis

Tumor fragments from PDX models were grafted into the interscapular fat pad of 6-week-old female Swiss nude mice under avertin anesthesia. When tumors reached a volume of 60-200 mm^3^, mice were blindly assigned to control (vehicle, NaCl 0.9%) or treated groups (at least n = 9 per condition). Mice were treated intraperitoneally by carboplatin (ACCORD) at 66 mg / kg every three weeks and paclitaxel (KABI) at 12 mg / kg once a week. Tumor growth was evaluated by measuring two perpendicular diameters of tumors with a caliper twice a week. Individual tumor volumes were calculated as (V) = a × b^2^ / 2, with “a” being the major and “b” the minor diameter. For each tumor, the tumor volume at day n (V_n_) was reported as the initial volume at time of inclusion (V_0_) and expressed as relative tumor volume (RTV) according to the following formula: RTV = V_n_ / V_0_. The mean and SEM of RTV in the same treatment group were calculated, and growth curves were established as a function of time. The percent of change to baseline was calculated at the end of treatment per mouse in all PDX models analyzed using the following formula: (RTV from carboplatin or carboplatin + paclitaxel treated mice/RTV from control mice) - 1 × 100. Baseline is the mean of the control group of mice. Studies were performed in compliance with protocol and animal housing in accordance with national regulation and international guidelines and under the supervision of authorized investigators. The experimental protocol and animal housing were in accordance with institutional guidelines as put forth by the French Ethical Committee (Agreement C75-05 - 18, France).

##### Protein Extraction

The same protocol was used for PDX and HGSOC samples. In brief, proteins were extracted using boiling lysis buffer (50 mM Tris pH 6.8, 2% SDS, 5% glycerol, 2 mM DTT, 2.5 mM EDTA, 2.5 mM EGTA, 4 mM Na3VO4 and 20 mM NaF) supplemented with 2 x Halt Phosphatase inhibitor (Perbio #78420) and complete EDTA-free protease inhibitor cocktail tablet (Roche #1836170). The protein extract was snap frozen in liquid nitrogen and stored at −80°C.

Grafting experiments were performed by subcutaneous injection of 2 × 10^6^ exponentially growing OC314-derived stable cell lines shCTRL, shPML#2 into one flank of 6-week-old female Swiss nude mice (at least 3 mice per group). Tumor growth was evaluated twice a week for 3 weeks.

### Quantification and Statistical Analysis

All statistical analyses were performed in the R environment (https://cran.r-project.org, Versions 3.3.2 and 3.4.0) or using GraphPad Prism software (version 7.0b). Data shown in this paper are generally represented as mean ± SEM from at least three independent experiments, unless otherwise specified. Statistical tests used are in agreement with data distribution: Normality was first checked using the Shapiro–Wilk test and parametric or non-parametric two-tailed tests were applied according to normality. Statistical tests used have been indicated in the legends of the figures. Spearman’s correlation test was used to evaluate the correlation coefficient between two parameters. Fisher’s exact test was used to determine an association between classes of ovarian cancers and clinical parameters. To assess biological interpretation of the most differentially expressed metabolic proteins, Gene ontology (GO) enrichment analysis was performed using the DAVID bioinformatics resources (https://david.ncifcrf.gov, Version 6.7). In order to avoid redundancy into GO terms and summarize information, we used the REViGO (Reduce and Visualize Gene Ontology) software (http://revigo.irb.hr, accessed January 2017), with a parameter similarity of 0.5. The optimal classification of HGSOC (from Curie and TCGA cohorts) was assessed by consensus clustering method (Monti et al., 2003) using the following parameters: clustering method: K-means, 1000 iterations, 80% of sample resampling. Survival analyses were carried out using Kaplan-Meier curves and p values were computed by Log-Rank test using survival R package. Stratification of patients for Kaplan-Meier analyses were performed using successive iterations to find the optimal sample size thresholds. Differences were considered to be statistically significant at values of p ≤ 0.05. The cut-off value was thus defined as the one that maximally discriminates the 2 patient subsets in each cohort. Overall survival was defined as: date of last news – date of diagnosis. Relapse at 12 months was defined as: date of relapse (progression or metastasis) – date of the end of 1^st^ line of treatment. If the event appears before 12 months: relapse = yes, otherwise relapse = no.

### Data and Software Availability

The results shown here are in part based upon data generated by the TCGA Research Network and available in a public repository from the https://cancergenome.nih.gov/ website. The authors declare that all the other data supporting the findings of this study are available within the article and its Supplemental Information files and from the corresponding author upon reasonable request. Original and analyzed data have been deposited through Mendeley data website under https://doi.org/10.17632/fstsb2xfsf.1.

## Supplemental Information

Document S1. Figures S1–S5 and Tables S1 and S3

Table S2. List of Metabolic Proteins Differentially Expressed in High- and Low-OXPHOS HGSOCs, Related to Figure 1 List of proteins differentially expressed in high- and low-OXPHOS HGSOC, derived from Possemato et al. (2011) and analyzed in Figure 1. Proteins are named by uniprot and gene symbols. p values are calculated from Mann-Whitney test, adjusted p value is from Benjamini-Hochberg procedure to account for multiple comparison testing. Fold change: ratio between the mean of the high-OXPHOS tumors (N = 74) and the mean of low-OXPHOS (N = 53) tumors.

Table S4. List of All Detected Proteins Detected in High- and Low-OXPHOS HGSOCs from the Curie Cohort, Related to Figure 1

Table S5. List of All Detected Metabolites Detected in High- and Low-OXPHOS HGSOCs from the Curie Cohort, Related to Figure 1

Document S1. Article plus Supplemental Information
