# Supplementary material for: PML-Regulated Mitochondrial Metabolism Enhances Chemosensitivity in Human Ovarian Cancers
Source: Cell Metab. 2019 Jan 8;29(1):156–173.e10. doi: 10.1016/j.cmet.2018.09.002 (PMC6331342; doi:10.1016/j.cmet.2018.09.002)
Supplement: Document S1. Figures S1–S5 and Tables S1 and S3 [file mmc1.pdf]

Cell Metabolism, Volume *28*

Supplemental Information

PML-Regulated Mitochondrial Metabolism Enhances Chemosensitivity in Human Ovarian Cancers

Géraldine Gentric, Yann Kieffer, Virginie Mieulet, Oumou Goundiam, Claire Bonneau, Fariba Nemati, Ilse Hurbain, Graca Raposo, Tatiana Popova, Marc-Henri Stern, Valérie Lallemand-Breitenbach, Sebastian Müller, Tatiana Cañeque, Raphaël Rodriguez, Anne Vincent-Salomon, Hugues de Thé, Rodrigue Rossignol, and Fatima Mechta-Grigoriou

SUPPLEMENTAL INFORMATION for:

PML-regulated mitochondrial metabolism enhances chemosensitivity in human ovarian cancers

Géraldine Gentric[1,2*], Yann Kieffer[1,2], Virginie Mieulet[1,2], Oumou Goundiam[1,2], Claire Bonneau[1,2], Fariba Nemati[3], Ilse Hurbain[4,5,6], Graca Raposo[4,5,6], Tatiana Popova[2,7], Marc-Henri Stern[2,7], Valérie Lallemand-Breitenbach[8], Sebastian Müller[9], Tatiana Cañeque[9], Raphaël Rodriguez[9], Anne Vincent-Salomon[10], Hugues de Thé[8], Rodrigue Rossignol[11] and Fatima Mechta-Grigoriou[1,2,12*]

Lead Contact: Fatima Mechta-Grigoriou (ORCID Number: 0000-0002-3751-6989) E-mail address: fatima.mechta-grigoriou@curie.fr

Table S1. Relative to Figure 1. Comparative description of the clinical parameters of the Institut Curie and TCGA cohorts of HGSOC patients

		Curie<br>Mateescu, Nat.Med. 2011<br>Gruosso, Nat. Commun. 2016	TCGA<br>TCGA, Nature, 2011
**Total number of patient**		**127**	**169**
**OXPHOS status**	**Low**	53 (41.7%)	90 (53.3%)
	**High**	74 (58.3%)	79 (46.7%)
**Median age (years)**			
	**Median age**	61	59
	**Min-Max**	35-87	34-87
**Histotype**			
	**Serous**	127 (100%)	169 (100%)
**Figo substage**			
	**I**	11 (8.7%)	-
	**II**	10 (7.9%)	9 (5.3%)
	**III**	83 (65.4%)	107 (63.3%)
	**IV**	15 (11.8%)	24 (14.2%)
	**NA**	8 (6.3%)	29 (17.2%)
**Grade**			
	**High-grade (G2, G3)**	127 (100%)	138
	**G1**	-	-
	**NA**		31
**Ki-67**			
	**Median**	1.9	
	**Min-Max**	0-4	
**Mitotic index**			
	**Median**	50	
	**Min-Max**	10-185	
**Metabolic Syndrome**			
	**Diabetes**	3 (2.5%)	
	**High blood pressure**	22 (18.2%)	
	**Dyslipidemia**	14 (11.6%)	
	**Body Mass Index (>30)**	6 (5%)	
**Surgery**			
	**No macroscopic residual**	33 (26%)	17 (10.1%)
	**Macroscopic residual**	72 (56.7%)	103 (60.9%)
	**NA**	22 (17.3%)	49 (29%)
**Chemotherapy**			
	**Yes**	123 (96.9%)	
	**NA**	4 (3.1%)	
**Chemotherapeutic agents**			
	**Platinum salt**	25 (19.7%)	
	**Platinum salt + taxanes**	98 (77.2%)	
	**NA**	4 (3.1%)	
**Relapse at 12 months**			
	**Yes**	54 (42.5%)	
	**No**	69 (54.3%)	
	**NA**	4 (3.1%)	
**LST signature**			
	**Low**	31 (24.4%)	68 (40.2%)
	**High**	55 (43.3%)	97 (57.4%)
	**NA**	41 (32.3%)	4 (2.4%)
**NtAi signature**			
	**Low**	21 (16.5%)	76 (45%)
	**High**	34 (26.8%)	88 (52%)
	**NA**	72 (56.7%)	5 (3%)
**HRD score**			
	**Low**	24 (18.9%)	71 (42%)
	**High**	31 (24.4%)	93 (55%)
	**NA**	72 (56.7%)	5 (3%)

**Patient**<br>Institut Curie	**HRD Status**	**Chemotherapy**	**PDX model**	**HRD Status**	**Chemotherapy**
Patient 1	HRD		OV5	HRD	
Patient 2	HRD	Sensitive	OV10	HRD	Sensitive
Patient 3	HRP	Sensitive	OV16	HRP	Sensitive
Patient 4	HRP	Resistant	OV26	HRP	Sensitive
Patient 5	HRD		OV8	NA	
Patient 6	HRP	Resistant	OV14	HRP	Resistant
Patient 7	HRP	Resistant	OV21	HRP	Resistant
Patient 8	HRD	Sensitive	OV25	HRD	Sensitive
Patient 9	HRD	Sensitive	OV38	HRD	Sensitive
Patient 10	HRD		OV54	HRD	

Table S1. Relative to Figure 1. Comparative description of the clinical parameters of the Institut Curie and TCGA cohorts of HGSOC patients

Top, Clinical parameters from Institut Curie and TCGA cohorts used for proteomic and metabolomic. TCGA and Curie cohorts have previously been described (Cancer Genome Atlas Research, 2011; Gruosso et al., 2015; Mateescu et al., 2011). Tumor samples were obtained from a cohort of consecutive ovarian carcinoma patients, treated at the Institut Curie between 1989 and 2012. All analyzed samples have been collected prior to any chemotherapeutic treatment. For each patient, a surgical specimen was taken before chemotherapy for pathological analysis and tumor tissue cryopreservation. The median patient age was 61 years (range: 35-87 years). Ovarian carcinomas were classified according to the World Health Organization histological classification of gynecological tumors. Pathological analysis identified 127 high-grade serous tumors (100%). 21 subjects (16.6%) were considered as early stage (International Federation of Gynecology and Obstetrics (FIGO) I-II) and 98 subjects (77.2%) were considered as advanced stage (III and IV) of disease. Proliferation rate was assessed by pathologists using KI-67 and mitotic index. Metabolic syndromes such as diabetes, high blood pressure, dyslipidemia or high body mass index are indicated. Classification of ovarian carcinomas according to debulking efficiency was done into two subgroups: “no macroscopic residual disease” and “macroscopic residual disease“, this last group including patients with tumor residual disease of 1-10mm, 11-20mm and > 20mm, after surgery. Patients were treated with a combination of surgery and chemotherapy, the latter including platinum salts or platinum salts ± taxane, as a first line of treatment in most cases. Relapse at 12 months was defined as: date of relapse (progression or metastasis) – date of the end of 1st line of treatment. If the event appears before 12 months: relapse = yes, otherwise relapse = no. Genomic signatures reflecting the HR status was assessed by using three scores : LST, N_{tAi} and HRD.

Bottom, ovarian cancer Patient Derived Xenograft (PDX) mouse models fully recapitulate histopathological and molecular properties, such as HRD status, as well as response to chemotherapy (86% of correspondence) of the patient tumors from which they derived.

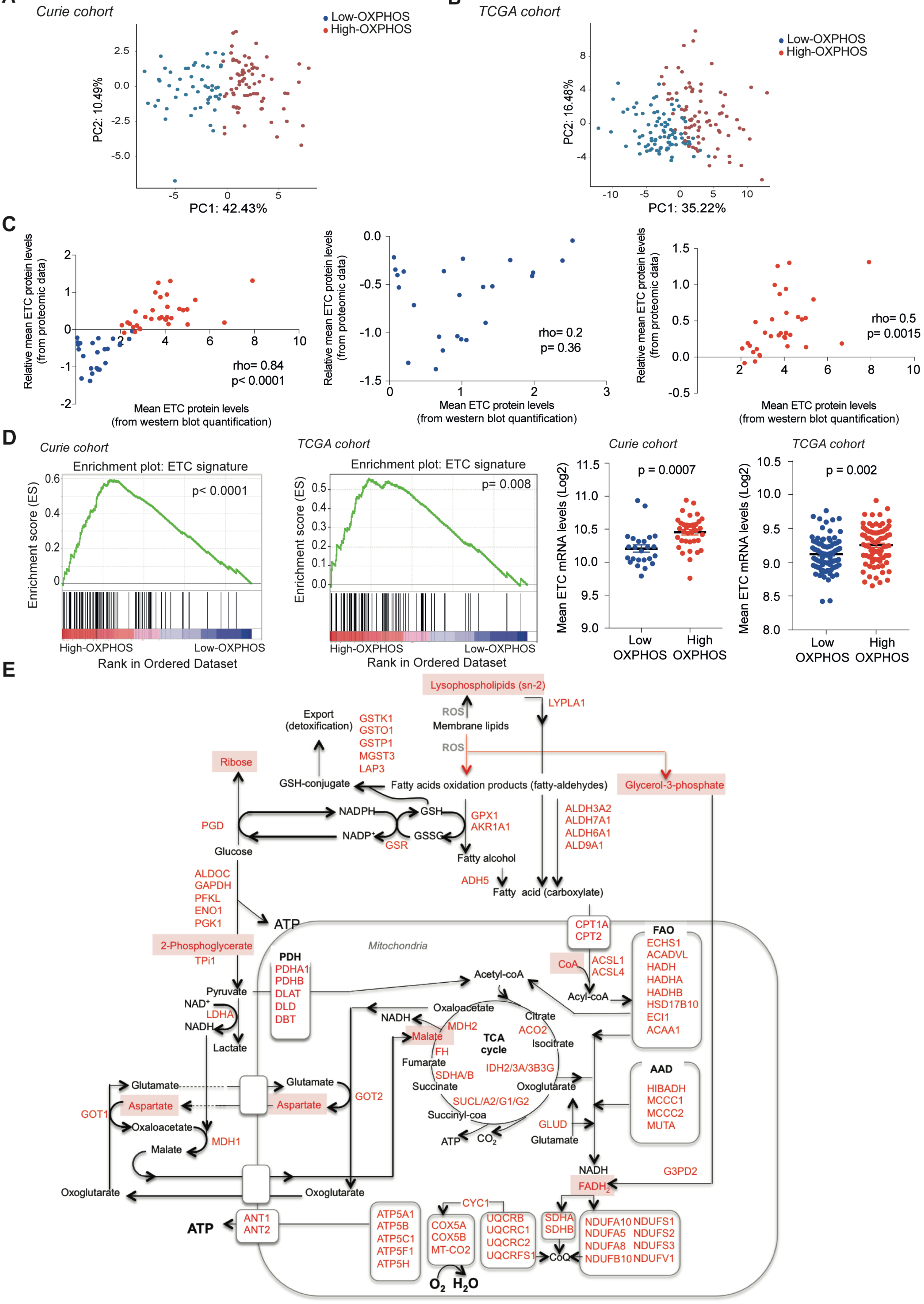
A
Curie cohort
Low-OXPHOS
High-OXPHOS
PC2: 10.49%
PC1: 42.43%
B
TCGA cohort
Low-OXPHOS
High-OXPHOS
PC2: 16.48%
PC1: 35.22%
C
Relative mean ETC protein levels (from proteomic data)
Mean ETC protein levels (from western blot quantification)
rho= 0.84
p< 0.0001
rho= 0.2
p= 0.36
rho= 0.5
p= 0.0015
D
Curie cohort
Enrichment plot: ETC signature
Enrichment score (ES)
p< 0.0001
High-OXPHOS
Low-OXPHOS
Rank in Ordered Dataset
TCGA cohort
Enrichment plot: ETC signature
p= 0.008
Curie cohort
p = 0.0007
Mean ETC mRNA levels (Log2)
Low OXPHOS
High OXPHOS
TCGA cohort
p = 0.002
E
Lysophospholipids (sn-2)
LYPLA1
ROS
Membrane lipids
Export (detoxification)
GSTK1
GSTO1
GSTP1
MGST3
LAP3
Ribose
GSH-conjugate
Fatty acids oxidation products (fatty-aldehydes)
Glycerol-3-phosphate
NADPH
GSH
NADP+
GSSG
GSR
GPX1
AKR1A1
ALDH3A2
ALDH7A1
ALDH6A1
ALD9A1
PGD
Glucose
Fatty alcohol
ADH5
Fatty acid (carboxylate)
ALDOC
GAPDH
PFKL
ENO1
PGK1
ATP
2-Phosphoglycerate
TPi1
Mitochondria
CPT1A
CPT2
FAO
ECHS1
ACADVL
HADH
HADHA
HADHB
HSD17B10
ECI1
ACAA1
CoA
ACSL1
ACSL4
Acyl-coA
PDH
PDHA1
PDHB
DLAT
DLD
DBT
Pyruvate
NAD+
LDHA
NADH
Lactate
Acetyl-coA
Oxaloacetate
Citrate
ACO2
MDH2
Malate
FH
TCA cycle
Isocitrate
Fumarate
IDH2/3A/3B3G
SDHA/B
Succinate
Oxoglutarate
SUCL/A2/G1/G2
Succinyl-coa
GLUD
Glutamate
CO2
AAD
HIBADH
MCCC1
MCCC2
MUTA
Glutamate
Aspartate
GOT1
GOT2
Oxaloacetate
MDH1
Malate
Oxoglutarate
NADH
G3PD2
FADH2
CYC1
ANT1
ANT2
ATP5A1
ATP5B
ATP5C1
ATP5F1
ATP5H
COX5A
COX5B
MT-CO2
UQCRB
UQCRC1
UQCRC2
UQCRFS1
SDHA
SDHB
CoQ
NDUFA10
NDUFA5
NDUFA8
NDUFB10
NDUFS1
NDUFS2
NDUFS3
NDUFV1
O2
H2O

Figure S1. Related to Figure 1. Identification of two OXPHOS subgroups in HGSOC

(**A**, **B)** Principal Component Analysis (PCA) applied on HGSOC proteomic data using ETC protein levels from Curie (N = 127 HGSOC) (**A**) and TCGA (N = 169 HGSOC) (**B**) cohorts. In Curie cohort, ETC protein levels have been standardized (centered and reduced) for each tumor sample. Normalization of TCGA data is described previously (Zhang et al., 2016). Low- and high-OXPHOS HGSOC are shown in blue and red, respectively. (**C**) Scatter plot showing positive correlation between the relative mean of 27 ETC protein levels defined by proteomic data (x axis) and of 5 ETC protein levels determined by western blot analysis (y axis) in low- (blue) and high- (red) OXPHOS (N = 58 HGSOC) (**Left**), restricted to low- (**Middle**) or to high- (**Right**) OXPHOS. *P*-value is from Spearman test. (**D) Left,** Gene set-enrichment analysis showing enrichment plot of ETC genes in Curie (**left**) and TCGA (**right**) cohort, as indicated. ETC genes (listed in **Table S3**) showed significant enrichment in high-OXPHOS HGSOC versus low-OXPHOS HGSOC. *P*-values are from false discovery rate (FDR) q-value. **(Right)** Scatter plots showing the relative mean levels of 96 ETC mRNA expression in Curie (**Left,** N = 60) and TCGA (**Right**, N = 169) cohorts. Data are shown as mean ± s.e.m. *P*-values are from Student's t-test. Low- (blue) and high- OXPHOS (red) HGSOC subgroups were defined according to the consensus clustering method. (**E**) Schematic representation of differential analyses of proteomic and metabolomic data from low- and high-OXPHOS HGSOC (Curie cohort). Represented are the major components of the metabolic rewiring observed in HGSOC, including glycolysis, fatty acid oxidation (FAO), reactive oxygen species detoxification, Amino Acid Degradation (AAD), malate-aspartate shuttle, the Tri-Carboxylic Acid (TCA) cycle and Electron Transport Chain (ETC) complexes. Proteins up-regulated in high- compared to low-OXPHOS HGSOC are written in red capital letters. Metabolites found in greater abundance in high- compared to low-OXPHOS HGSOC are shown in red boxes.

Table S3. Related to Figure S1, 3 and S3. List of ETC and NFE2L2-dependent antioxidant genes

ETC gene list		NFE2L2-antioxidant gene list	
MT-ND1	COX5B	ABCB6	PNKP
MT-ND2	COX6A1	ABCG2	POLRMT
MT-ND3	COX6A2	ABHD14B	PPIF
MT-ND4	COX6B1	ALDH16A1	PRDX6
MT-ND4L	COX6B2	ALDH3A2	PWP2H
MT-ND5	COX6C	AMID	RDM1
MT-ND6	COX7A1	ANKRD39	SEPP1
NDUFA1	COX7A2	ASL	SFTPD
NDUFA2	COX7B	ATOX1	SH3BP5
NDUFA3	COX7B2	ATP5L	SIRT2
NDUFA5	COX7C	ATPAF1	SLC19A1
NDUFA6	COX8A	BLVRB	SLC5A6
NDUFA7	COX8C	C5ORF23	SLC7A11
NDUFA8	MT-CO1	CA2	SMURF1
NDUFA9	MT-CO2	CAV2	SOD1
NDUFA10	MT-CO3	CBR1	SOD2
NDUFA11	ATP5A1	CCL5	SPPL2B
NDUFA12	ATP5B	CCNE2	SRXN1
NDUFA13	ATP5C1	CLCC1	THEM2
NDUFAB1	ATP5D	DEPDC1B	TREX1
NDUFB1	ATP5E	DHCR24	TTN
NDUFB2	ATP5F1	DLAT	TXN
NDUFB3	ATP5G1	EPHX1	TXNRD1
NDUFB4	ATP5G2	EPHX2	UCP2
NDUFB5	ATP5G3	EXOSC3	VLDLR
NDUFB6	ATP5H	FAH	ZDHHC21
NDUFB7	ATP5I	FBXO32	ZNF467
NDUFB8	ATP5J	GCLM	
NDUFB9	ATP5J2	GCNT2	
NDUFB10	ATP5L	GPD2	
NDUFB11	ATP5L2	GPT2	
NDUFC1	ATP5O	GPX1	
NDUFC2	ATPIF1	GPX2	
NDUFS1	MT-ATP6	GPX3	
NDUFS2	MT-ATP8	GSR	
NDUFS3		GTF2H4	
NDUFS4		HMOX1	
NDUFS5		IL17D	
NDUFS6		ISOC1	
NDUFS7		LIAS	
NDUFS8		LTB4DH	
NDUFV1		MAPK9	
NDUFV2		MCOLN1	
NDUFV3		MFSD3	
SDHA		MRPL4	
SDHB		MTHFD2L	
SDHC		MYC	
SDHD		NCF1	
CYC1		NLN	
MT-CYB		NOS2	
UQCR10		NOX4	
UQCR11		NOX5	
UQCRB		NQO1	
UQCRC1		NQO2	
UQCRC2		OXR1	
UQCRFS1		OXSR1	
UQCRH		P2RY6	
UQCRQ		PBK	
COX4I1		PEX6	
COX4I2		PIR	
COX5A		PLAA	

Table S3. Related to Figure 1, 3 and S3. List of ETC and NFE2L2-dependent antioxidant genes

List of ETC-encoding genes and NFE2L2-target genes analyzed in Figure S3B, S3C and Figure 3D, respectively.

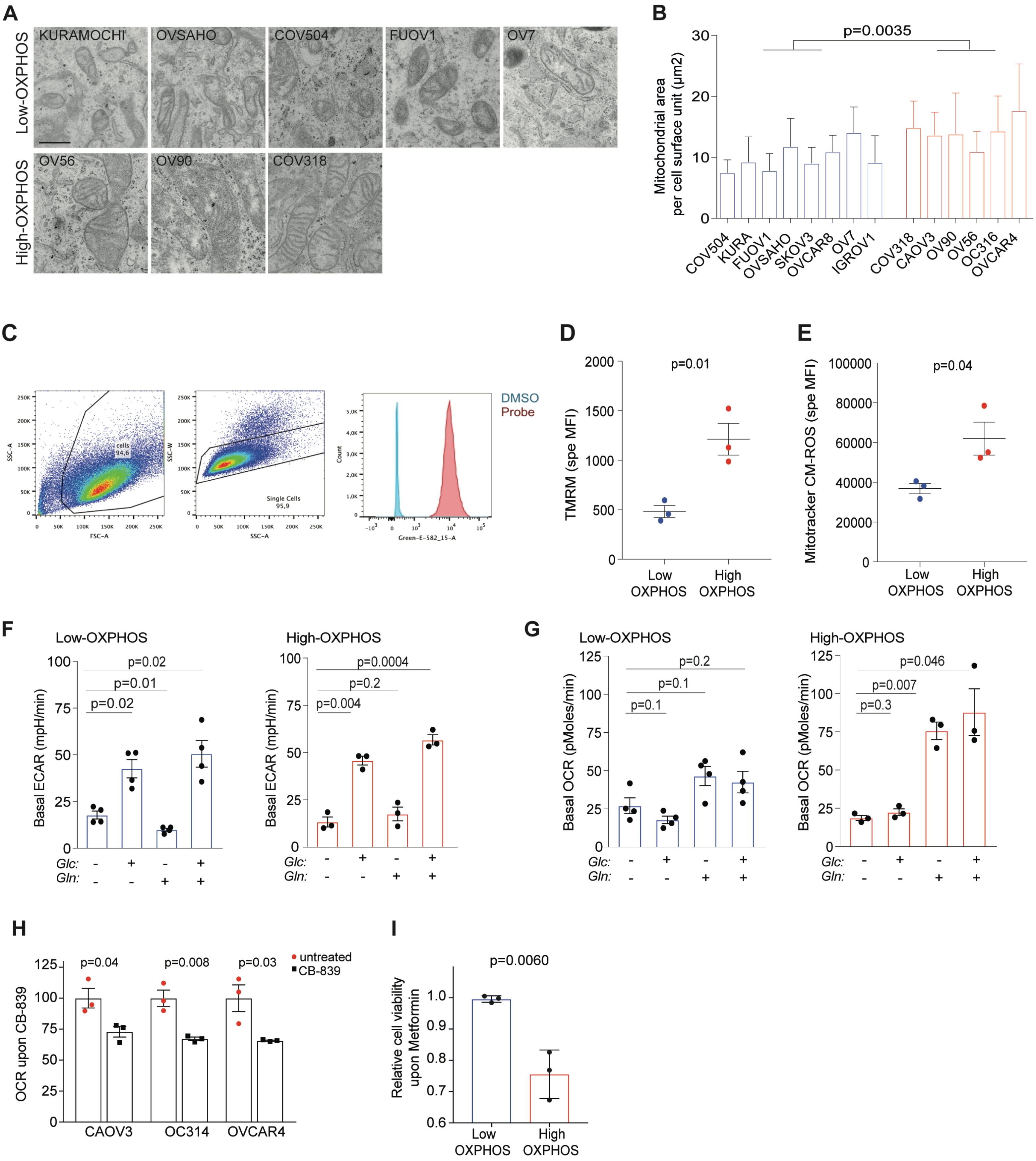
A
Low-OXPHOS
High-OXPHOS
KURAMOCHI
OVSAHO
COV504
FUOV1
OV7
OV56
OV90
COV318
B
p=0.0035
Mitochondrial area per cell surface unit (µm2)
30
20
10
0
COV504
KURA
FUOV1
OVSAHO
SKOV3
OVCAR8
OV7
IGROV1
COV318
CAOV3
OV90
OV56
OC316
OVCAR4
C
cells 94,6
Single Cells 95,9
FSC-A
SSC-A
SSC-W
Count
Green-E-582_15-A
DMSO
Probe
D
p=0.01
TMRM (spe MFI)
2000
1500
1000
500
0
Low OXPHOS
High OXPHOS
E
p=0.04
Mitotracker CM-ROS (spe MFI)
100000
80000
60000
40000
20000
0
Low OXPHOS
High OXPHOS
F
Low-OXPHOS
High-OXPHOS
Basal ECAR (mpH/min)
p=0.02
p=0.01
p=0.02
p=0.0004
p=0.2
p=0.004
100
75
50
25
0
Glc: - + - +
Gln: - - + +
G
Low-OXPHOS
High-OXPHOS
Basal OCR (pMoles/min)
p=0.2
p=0.1
p=0.1
p=0.046
p=0.007
p=0.3
125
100
75
50
25
0
Glc: - + - +
Gln: - - + +
H
OCR upon CB-839
p=0.04
p=0.008
p=0.03
untreated
CB-839
125
100
75
50
25
0
CAOV3
OC314
OVCAR4
I
p=0.0060
Relative cell viability upon Metformin
1.0
0.9
0.8
0.7
0.6
Low OXPHOS
High OXPHOS

Figure S2. Related to Figure 2. Mitochondrial characterization in low- and high-OXPHOS OCCL

(**A**) Representative pictures of electron microscopy from low- and high-OXPHOS OCCL, as indicated. Scale bar = 0.5 µm. (**B**) Bar plot showing mitochondrial area per unit of cell surface (in µm), as assessed using electron microscopy, in low- (blue) and high- (red) OXPHOS OCCL. Data are shown as mean ± s.e.m (n ≥ 8 pictures of electron microscopy analyzed per cell line). *P*-values are from Student's t-test. (**C**) Gating strategy used to measure specific MFI of Mitotracker and TMRM dyes. Debris and doublets were excluded based on SSC-A (side scatter), by FSC-A (forward scatter) parameters, and SSC-A by SSC-W parameters, respectively. DMSO was included as a negative control. (**D, E**) Scatter plots of specific MFI of TMRM (D) Mitotracker CM-ROS (E) dyes in low-(IGROV1, SKOV3, OVCAR8) and high-(CAOV3, OC314, OVCAR4) OXPHOS OCCL. Data are shown as mean ± s.e.m (n = 3 independent experiments). *P*-values are from Student's t-test. (**F, G**) Bar plots showing basal Extracellular Acidification Rate (EACR) (**F**) and OCR (**G**) in the presence of 10 mM glucose (Glc) or 2mM glutamine (Gln) or in control conditions (no Glc no Gln; or in presence of 10mM Glc + 2mM Gln) in low-(IGROV1, SKOV3, OVCAR8, OV7) (**Left**) and high-(CAOV3, OC314, OVCAR4) (**Right**) OXPHOS OCCL. Cells were incubated overnight in specific carbon media, as indicated. Data are shown as mean ± s.e.m (n ≥ 3 independent experiments). Each dot represents the mean value obtained in at least 3 independent experiments per cell line. *P*-values are from paired t-test. (**H**) Bar plot showing basal OCR in high-OXPHOS cells (CAOV3, OC314, OVCAR4) upon CB-839 treatment (10 µM) in the presence of 10 mM glucose + 2 mM glutamine in high-OXPHOS OCCL (CAOV3, OC314, OVCAR4), normalized to the mean of untreated condition for each cell line. Data are means ± s.e.m (n = 3 independent experiments). *P*-values from Student's t-test. (**I**) Bar plot showing variation in relative cell viability of low-(IGROV1, SKOV3, OVCAR8) and high- (CAOV3, OC314, OVCAR4) OXPHOS OCCL after 4 days of Metformin [10^{-2}M] treatment. All data are relative to vehicle-treated controls. Data are shown as mean ± s.e.m (n = 3 independent experiments). *P*-values are from Student-t-test.

A

Curie Cohort

	Diabetes No	Yes	NA	
Low-OXPHOS	40 (75%)	1 (2%)	12 (23%)	53 (100%)
High-OXPHOS	63 (85%)	2 (3%)	9 (12%)	74 (100%)

p=1

	Dyslipidiemia No	Yes	NA	
Low-OXPHOS	35 (66%)	6 (11%)	12 (23%)	53 (100%)
High-OXPHOS	57 (77%)	8 (11%)	9 (12%)	74 (100%)

p=0.8

	High Blood Pressure No	Yes	NA	
Low-OXPHOS	32 (60%)	9 (17%)	12 (23%)	53 (100%)
High-OXPHOS	52 (70%)	13 (18%)	9 (12%)	74 (100%)

p=0.8

	Body Mass Index (>30) No	Yes	NA	
Low-OXPHOS	37 (70%)	3 (6%)	13 (25%)	53 (100%)
High-OXPHOS	60 (81%)	3 (4%)	11 (15%)	74 (100%)

p=0.7

B

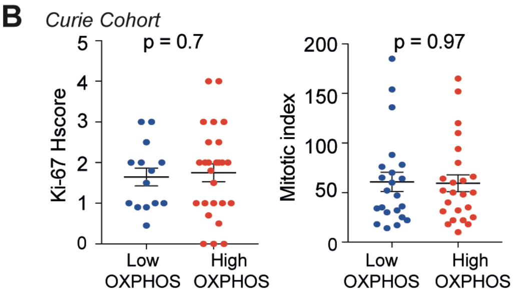

C

Curie Cohort

	Stages I-II	III-IV
Low-OXPHOS	7 (13%)	46 (87%)
High-OXPHOS	15 (20%)	59 (80%)

p=0.35

D

Curie Cohort

	Debulking No macroscopic residual	Macroscopic residual
Low-OXPHOS	14 (31%)	31 (69%)
High-OXPHOS	19 (32%)	41 (68%)

p=1

E **F** **G**

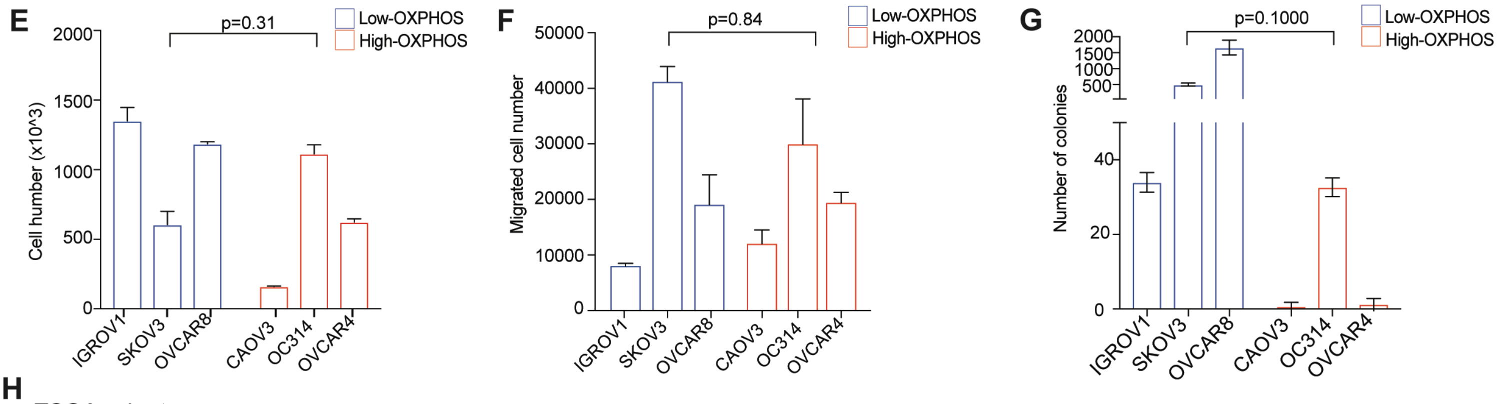

H

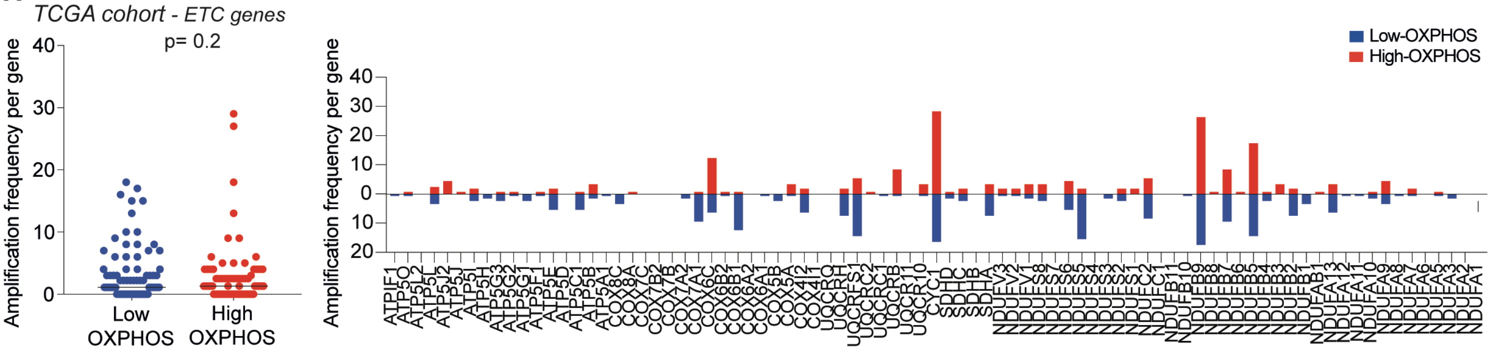

I

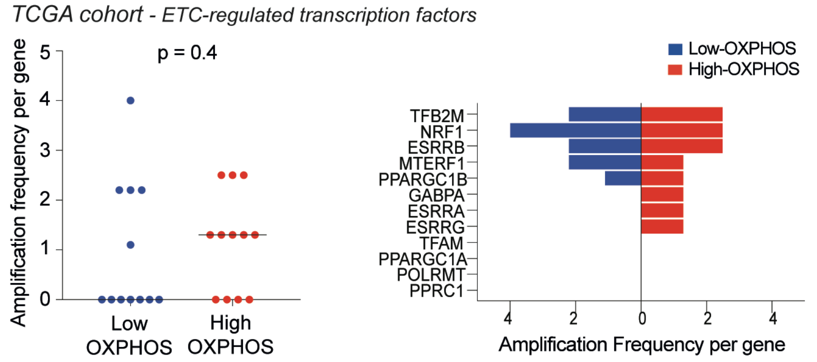

J

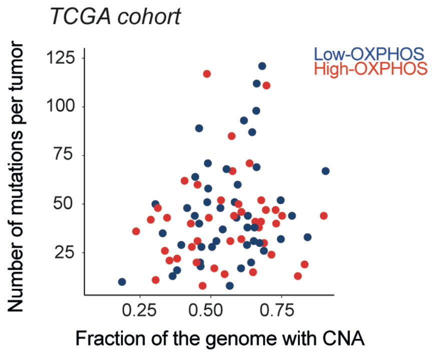

K

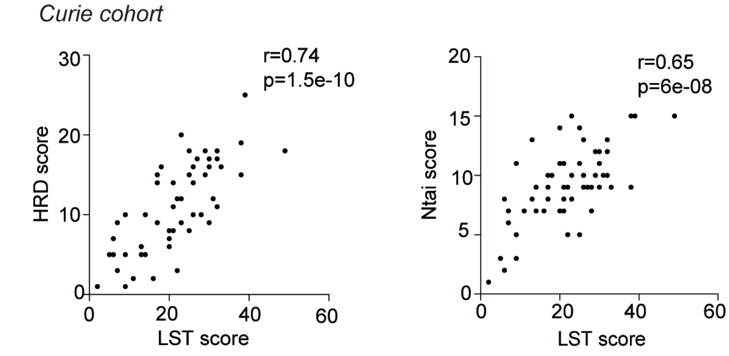

L

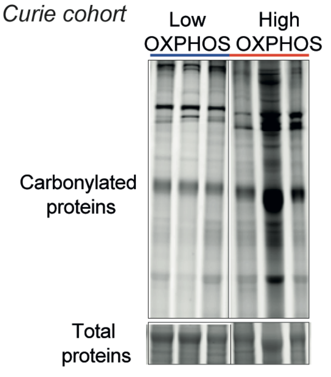

Figure S3. Related to Figure 3. Analyses of cellular properties and genomic alterations in high-OXPHOS HGSOC

(**A**) Contingency tables showing the repartition of low- and high-OXPHOS HGSOC from Curie cohort (N = 127) according to metabolic syndromes, including diabetes, dyslipidemia, high blood pressure and body mass index higher than 30. *P*-values are from Fisher's exact test. (**B**) Scatter plot showing Ki-67 (**Left**) and mitotic index (**Right**) in low- (blue) and high- (red) HGSOC from Curie cohort (N = 46). *P*-values are from Student-t-test. (**C, D**) Contingency tables showing the repartition of low- and high-OXPHOS HGSOC from Curie cohort (N = 127) according to stages (C) and debulking status (D). *P*-values are from Fisher's exact test. (**E**) Bar plot showing the cell number in low- (IGROV1, SKOV3, OVCAR8) and high- (CAOV3, OC314, OVCAR4) OXPHOS OCCL after 7 days of culture. Data are shown as mean ± s.e.m (n = 2 independent experiments). *P*-values are from Student-t-test. (**F**) Bar plot showing cell migration number in low- (IGROV1, SKOV3, OVCAR8) and high- (CAOV3, OC314, OVCAR4) OXPHOS OCCL. Data are shown as mean ± s.e.m (n = 2 independent experiments). *P*-values are from Student-t-test. (**G**) Bar plot showing the number of colonies in low- (IGROV1, SKOV3, OVCAR8) and high- (CAOV3, OC314, OVCAR4) OXPHOS OCCL. Data are shown as mean ± s.e.m (n = 3 independent experiments). *P*-values are from Student-t-test. (**H**) **Left**, Scatter plot showing amplification frequency per ETC gene in low- (blue) (N=88) and high- (red) (N=79) OXPHOS HGSOC from TCGA cohort. The median is indicated. *P*-values are from Wilcoxon test. **Right**, Bar plot showing amplification frequency per ETC gene, as on left. (**I**) **Left**, Scatter plot showing amplification frequency per gene encoding ETC-related transcription factor (listed on the right) in low- (blue) (N=88) and high- (red) (N=79) OXPHOS HGSOC from TCGA cohort. The median is indicated. *P*-values are from Wilcoxon test. **Right**, Bar plot showing amplification frequency per ETC-related transcription factor gene, as on left. (**J**) Plot showing absence of association between OXPHOS status (low-OXPHOS in blue, N = 46 and high-OXPHOS in red, N = 43) and mutation counts or copy number alterations (CNA) per tumor from TCGA cohort. (**K**) Plot showing the correlation between LST score and HRD score (Left) or N_{tai}

score (Right) in HGSOC from Curie cohort (N = 55). *P*-values are from Spearman test. (**L**) Western blot showing carbonylated protein profiles in 3 representative low- and high OXPHOS HGSOC (**Top**). SyproRuby coloration is used as control for total protein levels (**Bottom**).

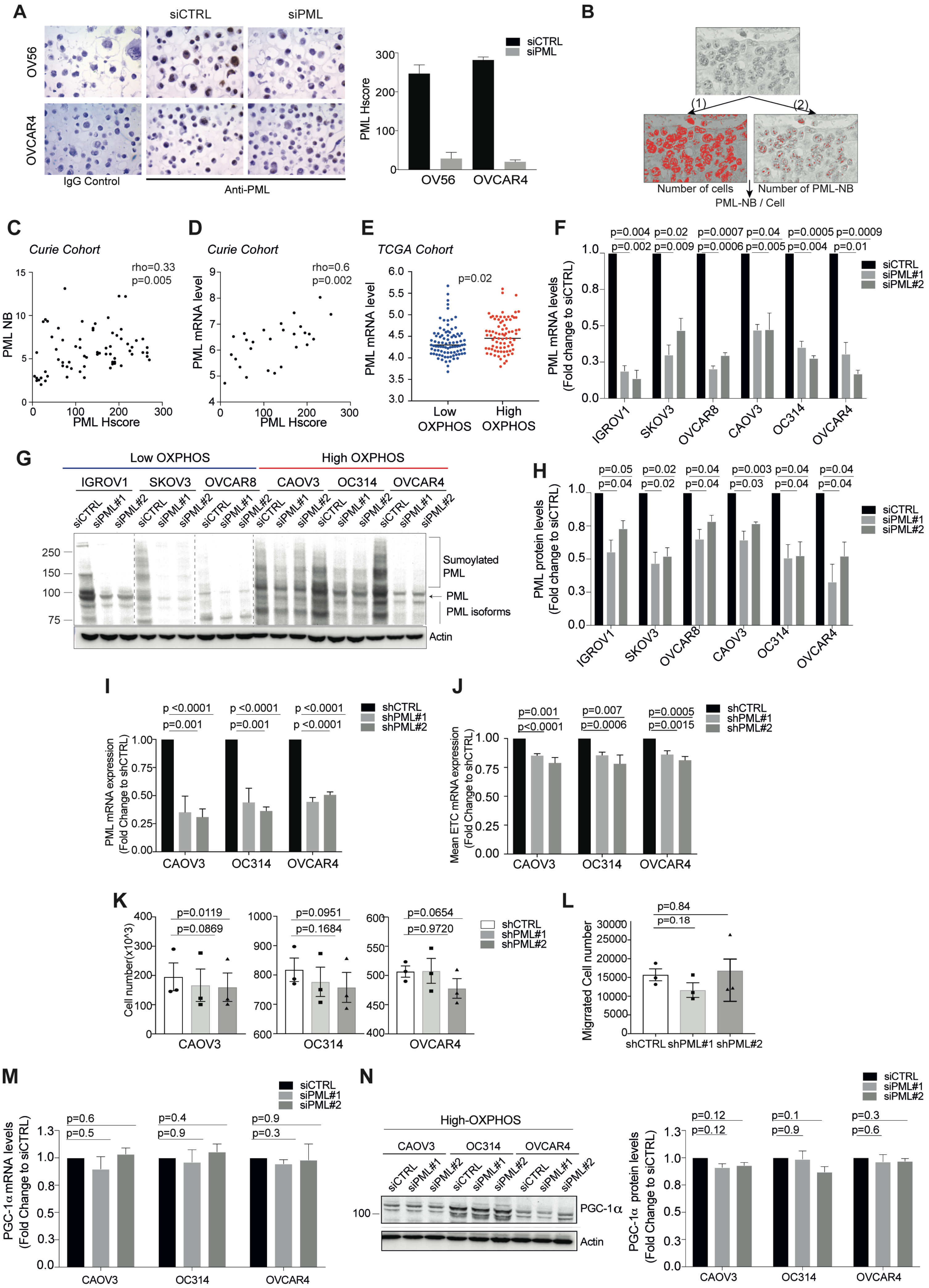
A
siCTRL
siPML
OV56
OVCAR4
IgG Control
Anti-PML
siCTRL
siPML
PML Hscore
OV56
OVCAR4
B
(1)
(2)
Number of cells
Number of PML-NB
PML-NB / Cell
C
Curie Cohort
rho=0.33
p=0.005
PML NB
PML Hscore
D
Curie Cohort
rho=0.6
p=0.002
PML mRNA level
PML Hscore
E
TCGA Cohort
p=0.02
PML mRNA level
Low OXPHOS
High OXPHOS
F
p=0.004 p=0.02 p=0.0007 p=0.04 p=0.0005 p=0.0009
p=0.002 p=0.009 p=0.0006 p=0.005 p=0.004 p=0.01
PML mRNA levels (Fold change to siCTRL)
siCTRL
siPML#1
siPML#2
IGROV1
SKOV3
OVCAR8
CAOV3
OC314
OVCAR4
G
Low OXPHOS
High OXPHOS
IGROV1
SKOV3
OVCAR8
CAOV3
OC314
OVCAR4
siCTRL siPML#1 siPML#2
Sumoylated PML
PML
PML isoforms
Actin
H
p=0.05 p=0.02 p=0.04 p=0.003 p=0.04 p=0.04
p=0.04 p=0.02 p=0.04 p=0.03 p=0.04 p=0.04
PML protein levels (Fold change to siCTRL)
siCTRL
siPML#1
siPML#2
IGROV1
SKOV3
OVCAR8
CAOV3
OC314
OVCAR4
I
p <0.0001 p <0.0001 p <0.0001
p=0.001 p=0.001 p <0.0001
shCTRL
shPML#1
shPML#2
PML mRNA expression (Fold Change to shCTRL)
CAOV3
OC314
OVCAR4
J
p=0.001 p=0.007 p=0.0005
p<0.0001 p=0.0006 p=0.0015
shCTRL
shPML#1
shPML#2
Mean ETC mRNA expression (Fold Change to shCTRL)
CAOV3
OC314
OVCAR4
K
p=0.0119
p=0.0869
p=0.0951
p=0.1684
p=0.0654
p=0.9720
Cell number(x10^3)
shCTRL
shPML#1
shPML#2
CAOV3
OC314
OVCAR4
L
p=0.84
p=0.18
Migrated Cell number
shCTRL shPML#1 shPML#2
M
siCTRL
siPML#1
siPML#2
p=0.6 p=0.4 p=0.9
p=0.5 p=0.9 p=0.3
PGC-1α mRNA levels (Fold Change to siCTRL)
CAOV3
OC314
OVCAR4
N
High-OXPHOS
CAOV3
OC314
OVCAR4
siCTRL siPML#1 siPML#2
PGC-1α
Actin
siCTRL
siPML#1
siPML#2
p=0.12 p=0.1 p=0.3
p=0.12 p=0.9 p=0.6
PGC-1α protein levels (Fold Change to siCTRL)
CAOV3
OC314
OVCAR4

Figure S4. Related to Figure 4. PML: antibody specificity and silencing effect in OCCL

(**A**) **Left**: Representative views of PML immunostaining on sections from paraffin-embedded OCCL after transfection either with non-targeting siRNA (siCTRL) or with a pool of siRNA targeting PML in order to test the specificity of the antibody used. **Right:** Bar plot showing PML-silencing efficiency (Hscore = Intensity of staining x % of cells stained). (**B**) Macro steps in ImageJ software to determine PML-NB per cell. (**C**) Scatter plot showing positive correlation between PML Hscore and PML-NB in HGSOC (N=71 HGSOC). *P*-value is from Spearman test. (**D**) Scatter plot showing positive correlation between PML Hscore and PML mRNA level in HGSOC (N=27 HGSOC). *P*-value is from Spearman test. (**E**) Scatter plot showing PML mRNA levels from TCGA cohort. The median is indicated (N = 169 HGSOC). *P*-values are from Mann-Whitney test. (**F**) Bar plot showing the fold change of PML mRNA levels in low- and high-OXPHOS OCCL transfected either with non-targeting siRNA (siCTRL) or with 2 different siRNA targeting PML (siPML#1, #2). qRT–PCR data are shown as mean of fold change normalized to non-targeting siRNA ± s.e.m (n ≥ 3 independent experiments). *P*-values are from one sample t-test. (**G**) Representative WB showing PML protein levels 48 hours post-transfection either with non-targeting siRNA (siCTRL) or with 2 different siRNA targeting PML (siPML#1, #2). Actin is used as an internal control for protein loading and normalization. All samples were run together on the same gel, but we used different exposition to show better the PML silencing effect, as indicated by the dotted line **(H)** Bar plot showing the fold change of PML protein levels 48 hours post transfection normalized to siCTRL and assessed by quantification of WB, as shown in (G). OCCL are transfected with either non-targeting siRNA (siCTRL) or 2 different siRNA targeting PML (siPML#1, siPML#2). Data are shown as mean ± s.e.m (n = 3 independent experiments). *P*-values are from one sample t test. (**I**) Bar plot showing the fold change of PML mRNA levels in high-OXPHOS OCCL transfected either with non-targeting shRNA (shCTRL) or with 2 different shRNA targeting PML (shPML#1, #2). qRT–PCR data are shown as mean of fold change normalized to non-targeting shRNA ± s.e.m (n = 3 independent experiments). *P*-values are from one sample t-test. (**J**) Bar plot showing the fold change in mean ETC mRNA levels in high-

OXPHOS OCCL transfected either with non-targeting shRNA (shCTRL) or with 2 different shRNA targeting PML (shPML#1, #2). 5 ETC (ATP5A, UQCR2, SDHB, COXII, NDUFB8) mRNA were analyzed to determine the mean ETC mRNA level. qRT–PCR data are expressed as the mean fold change normalized to non-targeting shRNA (n = 3 independent experiments). *P*-values are from one sample t test. (**K**) Bar plot showing the cell number in high-OXPHOS OCCL (CAOV3, left; OC314, middle; OVCAR4, right) transfected either with non-targeting shRNA (shCTRL) or with 2 different shRNA targeting PML (shPML#1, #2) after 7 days of culture. Data are shown as mean ± s.e.m (n = 3 independent experiments). *P*-values are from Student-t-test. (**L**) Bar plot showing cell migration number in and high-(CAOV3, OC314, OVCAR4) OXPHOS OCCL transfected either with non-targeting shRNA (shCTRL) or with 2 different shRNA targeting PML (shPML#1, #2). Data are shown as mean ± s.e.m (n = 3 independent experiments). *P*-values are from Student-t-test. (**M**) Bar plot showing the fold change of PGC-1α mRNA levels in high-OXPHOS OCCL transfected either with non-targeting siRNA (siCTRL) or with 2 different siRNA targeting PML (siPML#1, #2). qRT–PCR data are normalized to non-targeting siRNA and shown as the mean of fold change ± s.e.m (n = 3 independent experiments). *P*-values are from one sample t-test. (**N**) **Left**, Representative WB showing PGC-1α protein level in high-OXPHOS OCCL transfected either with non-targeting siRNA (siCRTL) or with 2 different siRNA targeting PML (siPML#1, #2). Actin is used as an internal control for protein loading and normalisation. **Right**, Bar plot showing the fold change of PGC-1α protein level upon PML silencing, as assessed by densitometry analysis of WB, as shown in (N). Data are shown as mean ± s.e.m (n = 3 independent experiments). *P*-values are from one sample t test.

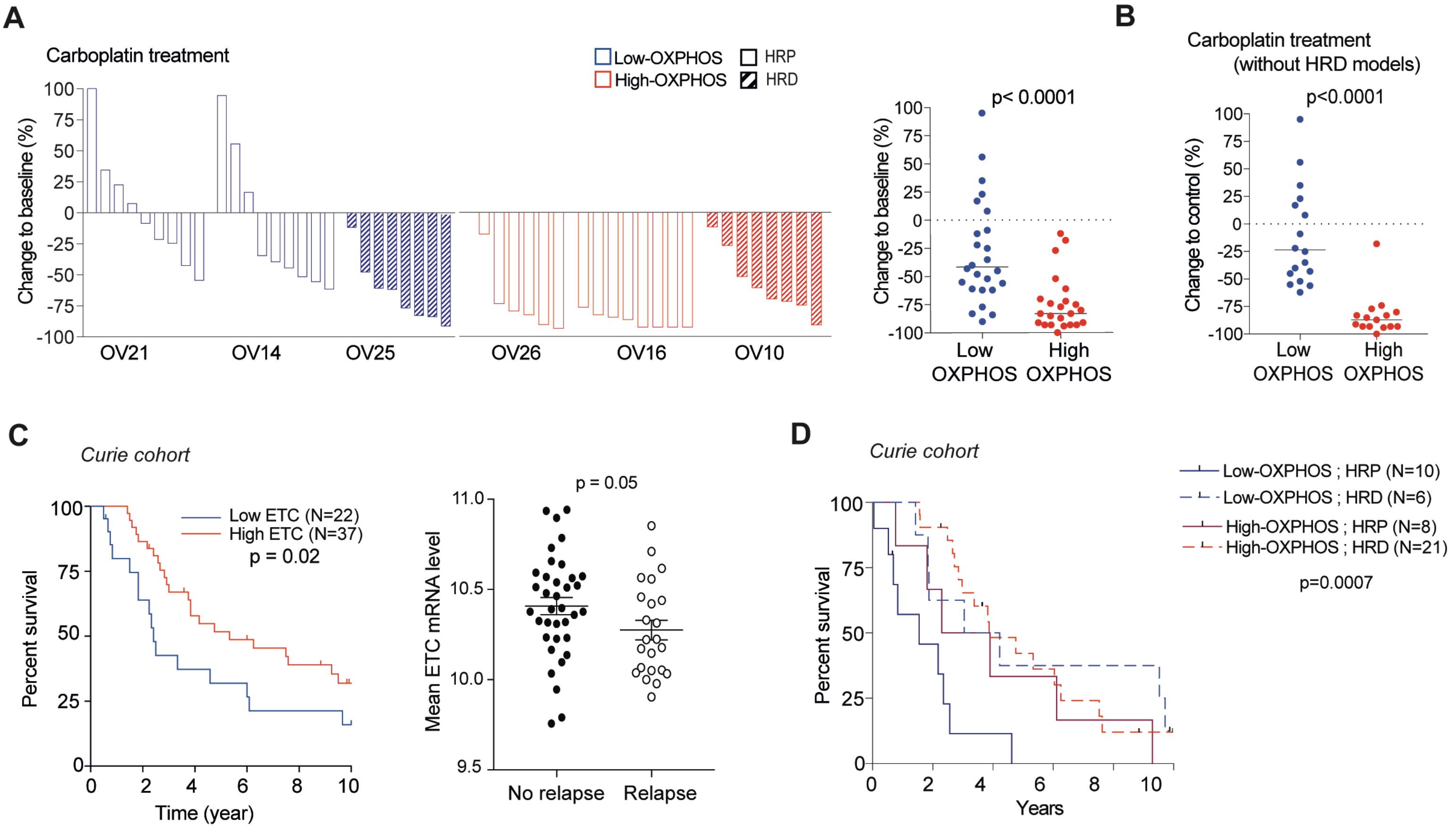

A
Carboplatin treatment
Low-OXPHOS
High-OXPHOS
HRP
HRD
Change to baseline (%)
OV21
OV14
OV25
OV26
OV16
OV10
p< 0.0001
Low OXPHOS
High OXPHOS
B
Carboplatin treatment (without HRD models)
p<0.0001
Change to control (%)
C
Curie cohort
Low ETC (N=22)
High ETC (N=37)
p = 0.02
Percent survival
Time (year)
p = 0.05
Mean ETC mRNA level
No relapse
Relapse
D
Curie cohort
Low-OXPHOS ; HRP (N=10)
Low-OXPHOS ; HRD (N=6)
High-OXPHOS ; HRP (N=8)
High-OXPHOS ; HRD (N=21)
p=0.0007
Percent survival
Years

Figure S5. Related to Figure 5. High-OXPHOS metabolism is associated with better survival and absence of relapse at 12 months in HGSOC patients

(A) Left, Waterfall plots showing individual percent change to baseline at the end of treatment per mouse in all PDX models analyzed following carboplatin treatment. Baseline is the mean of the control group of mice. Low- and high-OXPHOS HGSOC PDX models are shown in blue and red, respectively. The percent of change to baseline is calculated using the following formula: (RTV from carboplatin treated mice/RTV from control mice)-1 x 100. **Right**, Scatter plot of data on left combining the different PDX models and comparing low- and high-OXPHOS, as indicated. *P*-values are from Mann-Whitney test. The median is indicated. (**B**) Scatter plot combining the percentage of change to baseline from the PDX models excluding the HRD PDX models and comparing low- (OV21 and OV14) and high- (OV26 and OV16) OXPHOS, as indicated. *P*-values are from Mann-Whitney test. The median is indicated. (**C**) Kaplan-Meier curves showing 10-years overall survival of HRD and HRP patients from Curie cohort with HGSOC of low- (blue) or high- (red) ETC subgroups based on the mean ETC mRNA level. *P*-values are from Log-rank test. (**Right**) Scatter plot showing the mean ETC mRNA level per HGSOC from Curie cohort according to the relapse status at 12 months after the end of the first line of chemotherapy treatment. Data are shown as mean ± s.e.m. *P*-values are from Student's t-test. (**D**) Kaplan-Meier curves showing 10-years overall survival of patients from Curie cohort with HGSOC of low- (blue) or high- (red) OXPHOS subtypes and stratified in HRP (solid line) or HRD (dotted line) using the LST signature. $P = 0.0007$ from Log-rank test.
